# Apigenin Inhibits IL-6 Transcription and Suppresses Esophageal Carcinogenesis

**DOI:** 10.3389/fphar.2019.01002

**Published:** 2019-09-11

**Authors:** Jian-Ge Qiu, Lin Wang, Wen-Jing Liu, Ju-Feng Wang, Er-Jiang Zhao, Feng-Mei Zhou, Xiang-Bo Ji, Li-Hong Wang, Zhong-Kun Xia, Wei Wang, Marie Chia-mi Lin, Ling-Zhi Liu, Ying-Xue Huang, Bing-Hua Jiang

**Affiliations:** ^1^The First Affiliated Hospital of Zhengzhou University, Zhengzhou University, Zhengzhou, Henan, China; ^2^Department of Internal Medicine, Affiliated Cancer Hospital of Zhengzhou University, Zhengzhou, Henan, China; ^3^Department of Biostatistics, The Affiliated Cancer Hospital of Zhengzhou University, Zhengzhou, Henan, China; ^4^Department of Pathology, Carver College of Medicine, University of Iowa, Iowa, IA, United States

**Keywords:** apigenin, esophagus cancer, interleukin 6, tumor growth, inhibition

## Abstract

Esophagus cancer is the seventh cause of cancer-related deaths globally. In this study, we analyzed interleukin 6 (IL-6) gene expression in human esophagus cancer patients and showed that IL-6 mRNA levels are significantly higher in tumor tissues and negatively correlated with overall survival, suggesting that IL-6 is a potential therapeutic target for esophagus cancer. We further demonstrated that apigenin, a nature flavone product of green plants, inhibited IL-6 transcription and gene expression in human esophagus cancer Eca-109 and Kyse-30 cells. Apigenin significantly and dose-dependently inhibited cell proliferation and promoted apoptosis while stimulating the cleaved PARP (poly ADP-ribose polymerase) (C-PARP) and caspase-8 expression. It suppressed VEGF (Vascular endothelial growth Factor) expression and tumor-induced angiogenesis. Pretreatment of cells with IL-6 could completely reverse apigenin-induced cellular changes. Finally, using a preclinical nude mice model subcutaneously xenografted with Eca-109 cells, we demonstrated the *in vivo* antitumor activity and mechanisms of apigenin. Taken together, this study revealed for the first time that apigenin is a new IL-6 transcription inhibitor and that inhibiting IL-6 transcription is one of the mechanisms by which apigenin exhibits its anticancer effects. The potential clinical applications of apigenin in treating esophagus cancer warrant further investigations.

## Introduction

Esophagus cancer is the seventh cause of cancer-related deaths globally (GLOBOCAN, 2018) (Bollschweiler et al., 2017). This disease is curative in the early stage *via* surgery; however, most cases are diagnosed at the advanced stage and require systemic chemotherapy (Edwards et al., 2018). While systemic chemotherapy can significantly improve the survival and quality of life (Wagner et al., 2017), nevertheless, drug resistance is a major challenge (Bolm et al., 2018). Despite the development of multimodality therapies, including surgery combined with chemotherapy and/or radiotherapy, the prognosis of esophagus cancer patients remains poor (Nakajima and Kato, 2013). Discovery of new drug targets and more effective drugs is important for esophagus cancer treatment.

Interleukin 6 (IL-6) is a proinflammatory cytokine released by cells in the tumor microenvironment (Masjedi et al., 2018). Interleukin 6 plays critical roles in the differentiation and expansion of tumor cells. Elevated IL-6 level has been reported in breast (Guo et al., 2012; Dethlefsen et al., 2013), gastric, bile duct, pancreatic and colorectal cancer patients (Vainer et al., 2018). There is a published report that described elevated IL-6 level in the esophagus tumor tissue of a 51-year-old male patient, suggesting that IL-6 may have a role in esophagus tumor development (Shioga et al., 2018). At present, two monoclonal antibodies against IL-6, tocilizumab and siltuximab, have been shown to have antitumor activities (Yao et al., 2014; Ham et al., 2019; Weng et al., 2019). However, to our knowledge, no small molecule IL-6 inhibitor has been reported.

Apigenin (4′,5,7-trihydroxyflavone), a small molecule natural compound extracted from plants, belongs to the flavone class constituting the aglycone of various natural element glycosides. Numerous vegetables and fruits, such as celeriac, chamomile tea, and parsley, are rich in apigenin (Salmani et al., 2017). Apigenin has been shown to exhibit antitumor activity in lung, pancreatic, breast, hepatic, prostate, and colon cancers (Johnson and De Mejia, 2013; Pan et al., 2013; Shao et al., 2013; Lee et al., 2014; Shukla et al., 2014; Huang et al., 2016). At present, it is not known whether apigenin has therapeutic effect against esophageal cancer.

In this study, we demonstrated for the first time that apigenin is an IL-6 transcription inhibitor in human esophagus cancer Eca-109 and Kyse-30 cells. It inhibited the transcription and expression of IL-6. In addition, apigenin suppressed cell proliferation and tumor-induced angiogenesis and promoted apoptosis *in vitro* in Eca-109 and Kyse-30 cells. Pretreatment of Eca-109 and Kyse-30 cells with excess of IL-6 could completely reverse apigenin-induced cellular changes. Finally, we demonstrated the *in vivo* antitumor activity and mechanisms of apigenin in a preclinical nude mice model subcutaneously xenografted with Eca-109 cells. The potential clinical applications of apigenin in treating esophagus cancer warrant further investigations.

## Materials and Methods

### Reagents, Cell Lines, and Cell Culture

Human esophagus cancer Eca-109 and Kyse-30 cell lines were purchased from Procell Life Science & Technology Co., Ltd., and Cellcook Biotechnology Co., Ltd. Cells were cultured at 37°C in a humidified atmosphere of 5% CO_2_ in the RPMI1640 medium, supplemented with 10% fetal bovine serum and antibiotics (100 U/ml penicillin and 100 mg/ml streptomycin). Apigenin was obtained from Sigma-Aldrich and dissolved in dimethyl sulfoxide, stored at −20°C until ready for use. Growth factor–reduced Matrigel was from BD Biosciences (Bedford, MA, USA). The pIL-6-promoter-luc and Renilla-luc plasmids were purchased from Beyotime Biotechnology (Shanghai, China). The antibodies against PARP and caspase-8 were from Cell Signaling Technology (Beverly, MA, USA). Monoclonal antibodies against IL-6 and GAPDH were from Proteintech (Wuhan, China).

### Kaplan-Meier Analysis of Survival Probability and Gene Expression

RNAseq and clinical data were acquired from TCGA database; the information on esophagus cancer (*n* = 160) datasets was downloaded from the UCSC Xena Browser (https://xenabrowser.net/heatmap/#). Gene expression level and survival analysis were performed with Kaplan-Meier estimator and *post hoc* log-rank test using GraphPad software.

### Quantitative Real-Time Polymerase Chain Reaction

Quantitative real-time reverse transcriptase–polymerase chain reaction (PCR) was used to determine the mRNA expression levels of VEGF and GAPDH. Total RNAs were extracted by Trizol reagent; reverse transcriptions were performed using SYBR Premix Dimer Eraser according to the manufacturer’s instruction. Real-time PCR was performed using QuautStudio-5 Real-Time Thermal Cycler (ABI). The following primer sequences were used for PCR: VEGF forward primer: 5′-TGTCTAATGCCCTGGAGCCT-3′; reverse primer: 5′- GCTTGTCACATCTGCAAGTACG-3′; and GAPDH forward primer: 5′- ATGGGTGTGAACCATGAGAAGTATG-3′; reverse primer: 5′-GGTGCAGGAGGCATTGCT-3′. The expression levels of VEGF were normalized to the value of GAPDH, and fold changes were calculated by relative quantification (2^−^ΔΔCt).

### IL-6 Promoter Activity is Determined by the Luciferase Assay

The promoter sequence (−637 bp ∼ +53 bp) of human IL-6 was cloned into the pGL6 vector, to prepare the pIL-6-promoter-luc vector for IL-6 promoter activity measurement. Eca-109 and Kyse-30 cells were seeded into a 24-well plate, cultured overnight, cotransfected with pIL-6-promoter-luc and Renilla-luc plasmids, and then treated with different concentrations of apigenin. Firefly and Renilla luciferase activities were measured 48 h later by the Dual Luciferase Reporter Assay System (Promega, WI, USA) and normalized to the corresponding controls as described previously (Xu et al., 2012). The IL-6 promoter activity was calculated as the ratio of Firefly luciferase value/Renilla luciferase value. Experiments were performed in three independent replicates (Xu et al., 2013).

### Cell Proliferation Assay

To determine the effects of apigenin on the cell proliferation of esophagus cells, Eca-109 and Kyse-30 cells were seeded into 6-well plates at 3 × 10^5^ cells per well, cultured overnight, and then treated with experimental medium containing various concentrations of freshly prepared apigenin at the final concentrations ranging from 0.3 to 10 µM. After 48 h of treatment, cells were stained with crystal violet, and cell numbers were calculated. Data were from three separate experiments with four replications.

### Apoptosis Assay

Human esophagus cells were seeded into 6-well plates (3 × 10^5^ cells/well) and treated with apigenin (0 µM vehicle control; 0.3, 1, 3, and 10 µM), with or without IL-6 (50 ng/µl). After 48 h, cells were harvested and stained with annexin V–fluorescein isothiocyanate (FITC) and propidium iodide (PI) for 15 min in the dark. BD ACCURI C6 PLUS Cell Analyzer was used to measure fluorescence intensity in FITC (FL1, 533 nm) and PI (FL2, 585 nm) channels. The early apoptotic cells (annexin V-positive only) and late apoptotic cells (annexin V- and PI-positive) were quantified and analyzed with the FlowJo 10.0.7 software.

### Western Blot

Western blot was performed as described previously (Liu et al., 2011). Human esophagus cancer cells were harvested, washed with cold phosphate-buffered saline (PBS), and suspended in 100 µl of cold cell lyssis buffer with protease inhibitor. The lysates were incubated on ice for 30 min and centrifuged for 10 min at 4°C, supernatants collected, and the protein concentration quantified by Bradford method (Bradford, 1976). Cell lysates were resolved by sodium dodecyl sulfate–polyacrylamide gel electrophoresis and transferred onto polyvinylidene fluoride membranes and incubated in blocking buffer [5% bovine serum albumin in TBST (Tris-Buffered Saline Tween-20)] for 2 h at room temperature. The membranes were incubated with primer antibody overnight at 4°C, then horseradish peroxidase–conjugated secondary antibody for 1 h at room temperature, and washed three times with TBST. The protein-antibody complex was detected by a chemiluminescence detection system. The relative expression level of each protein in the experimental group was calculated against the control group (as one).

### Tube Formation Assay

To prepare for conditional medium, Eca-109 and Kyse-30 cells were seeded into a 24-well plate, cultured overnight, and treated with medium containing indicated concentrations of apigenin for 24 h. The medium of each group was then collected and stored at −20°C until ready for use. For tube formation assay, 50 µl Matrigel (BD Biosciences, San Jose, CA, USA) was added into each well of the 96-well plate and polymerized for 1 h at 37°C. Then, human umbilical vein endothelial cells (HUVECs) in 50 µl of various conditional medium were added to each well and incubated for 8 h, and the image of tube formation was captured with a digital camera. The capillary tubes were quantified by determination of length. Each condition was assessed in triplicate and repeated once.

### ELISA Assay

The levels of IL-6 in tumor tissues were detected by enzyme-linked immunosorbent assay (ELISA) kits following manufacturer’s instructions. The concentrations of IL-6 were measured using a microplate reader. Each condition was assessed in triplicate and repeated once.

### Nude Mice Xenografted With Human Esophagus Eca-109 Cell

Four-week-old male nude mice were purchased from the Beijing Vital River Laboratory Animal Technology Co., Ltd., maintained in pathogen-free conditions, and sustained with standard diets. All studies were approved by the Institutional Committee on Animal Care of Zhengzhou University. Eca-109 cells in 200 µl of PBS (3 × 10^6^ cells/tumor) were subcutaneously injected into nude mice on both sides of the armpit. When solid tumors grew to 0.5-cm diameter (5 days), mice were divided randomly into four groups (*n* = 6) and treated daily for 3 weeks by intraperitoneal injection, with either vehicle control (saline) or apigenin (1, 3, 5, and 10 mg/kg). Tumor volume was monitored daily and calculated using the formula: *V* = (π/6) × [(*A* + *B*)/2]^3^ (*A*, longest diameter; *B*, shortest diameter). Four weeks after drug treatment, mice were sacrificed, and tumors were dissected for immunohistochemistry and other analyses. The animal study was repeated once with similar results.

### Immunohistochemistry

Immunohistochemistry was performed as described previously (Xu et al., 2012). Tumors were harvested, fixed, paraffin embedded, and sectioned (8 µm). Tumor tissue slides were deparaffinized using xylene and graded ethyl alcohol and rinsed with water. Antigen retrieval was performed by boiling the slides in 0.01 M citrate buffer in a microwave oven for 10 min and cooling at room temperature. The slides were incubated with 0.05% Triton-X 100 in PBS for 5 min, followed by sequential treatment in a humidified chamber after quenching endogenous peroxides with 3% H_2_O_2_ in MeOH, and then blocked with serum for 20 min and incubated with anti-CD31 or anti-Ki67 antibody overnight at 4°C, then secondary antibody for 20 min, hydrogen peroxidase for 15 min, and peroxidase substrate solution for 20 min at room temperature. The stained slides were counterstained with hematoxylin and coverslipped. The percentages of CD31- and Ki67-positive cells were quantified as the average of five fields for each slide.

### Statistical Analysis

All results are expressed as mean ± standard deviation (SD), and all experiments were repeated at least three times. Statistical analyses were performed using Prism (GraphPad Software) by one‐way/two‐way analysis of variance with *post hoc* Bonferroni/Dunnett test or Student *t* test as indicated. Survival analyses were performed using Kaplan-Meier estimator with *post hoc* log‐rank test. The significance was indicated at *P* < 0.05; highly significant difference was indicated at *P* < 0.01.

## Results

### The Elevated Tissue and Plasma IL-6 Expression Levels in Human Esophagus Cancer Patients Are Negatively Correlated With the Overall Survival

**Figure 1:**
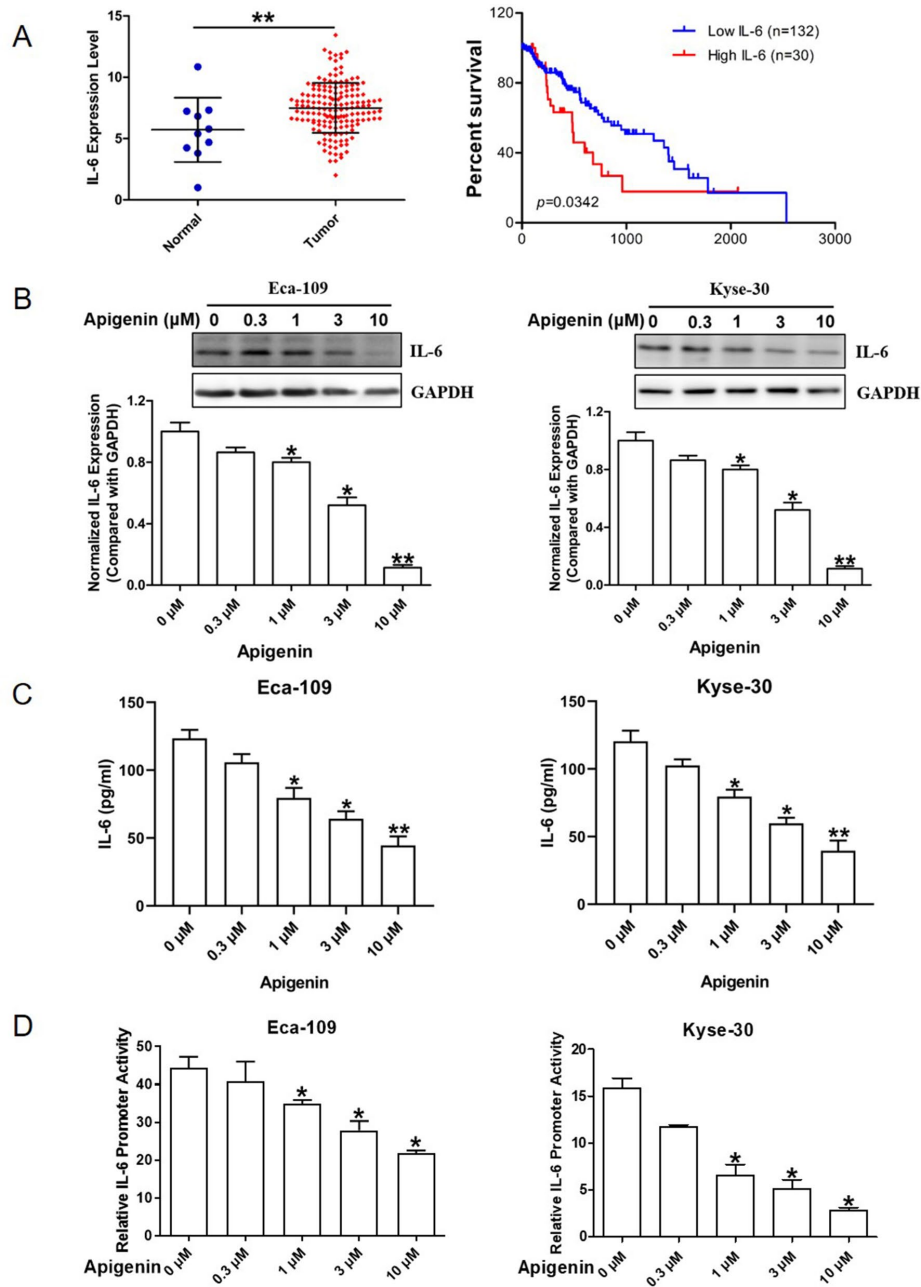
Elevated IL-6 expression and inhibition of IL-6 expression by apigenin in human esophagus cancer Eca-109 and Kyse-30 cells. **(A)** The IL-6 expression level in normal and tumor tissues of esophagus cancer patient samples (left panel) and the relationship between patient survival and IL-6 expression by Kaplan-Meier estimator with *post hoc* log-rank test (right panel). **(B)** Western blotting analysis of Eca-109 (left panel) and Kyse-30 cells (right panel) treated with various concentrations of apigenin. **(C)** ELISA analysis of the culture medium collected from Eca-109 (left panel) and Kyse-30 cells (right panel) treated with various concentrations of apigenin. **(D)** The relative luciferase activities in Eca-109 (left panel) and Kyse-30 cells (right panel) transiently transfected with IL-6 promoter luciferase reporter plasmid and then treated with the indicated concentrations of apigenin. *P < 0.05 indicates significant difference as compared to the corresponding control (*n* = 3). *P < 0.05 and **P < 0.01 indicate significant difference as compared to the corresponding control (*n* =3).

We examined the IL-6 gene expression data in human esophagus cancer using a publicly available database from TCGA network derived from the UCSC Xena Browser. In esophagus cancer patient samples, the IL-6 mRNA levels in tumor tissues were significantly higher than those in the adjacent normal tissues (**Figure 1A**, left panel). In addition, the increased plasma IL-6 mRNA level is negatively (*P* = 0.0342) correlated with overall survival (**Figure 1A**, right panel).

### Apigenin Treatment Reduced IL-6 Expression and the IL-6 Promoter Activity in Human Esophagus Cancer Eca-109 and Kyse-30 Cells

We first evaluated the effect of apigenin on the expression of IL-6 in human esophagus cancer Eca-109 and Kyse-30 cells. As shown in **Figure 1B**, Western blot analysis indicated that apigenin treatment down-regulated IL-6 protein expression level dose-dependently. Treatment of 0.3, 1, 3, and 10 µM of apigenin produced significant 10%, 20%, 50%, and 89% reductions of IL-6 protein levels in Eca-109 cells, respectively, as well as 13%, 23%, 56%, and 78% reductions in Kyse-30 cells, respectively. Apigenin treatment also significantly decreased the level of secreted IL-6 in the cell culture medium (**Figure 1C**). To investigate whether apigenin could down-regulate IL-6 expression by transcription inhibition, we prepared an IL-6 promoter reporter plasmid and determined IL-6 promoter activity as described in the Methods section. As shown in **Figure 1D**, 1, 3, and 10 µM of apigenin produced significant 22%, 40%, and 51% reductions of the IL-6 promoter activity in Eca-109 cells, respectively, as well as 60%, 68%, and 81% reductions, respectively, in Kyse-30 cells. Taken together, these results indicated that apigenin decreased IL-6 expression through inhibiting the transcription activity of IL-6 in esophagus cancer cells.

### Apigenin Inhibited Cell Proliferation in Eca-109 and Kyse-30 Cells

**Figure 2:**
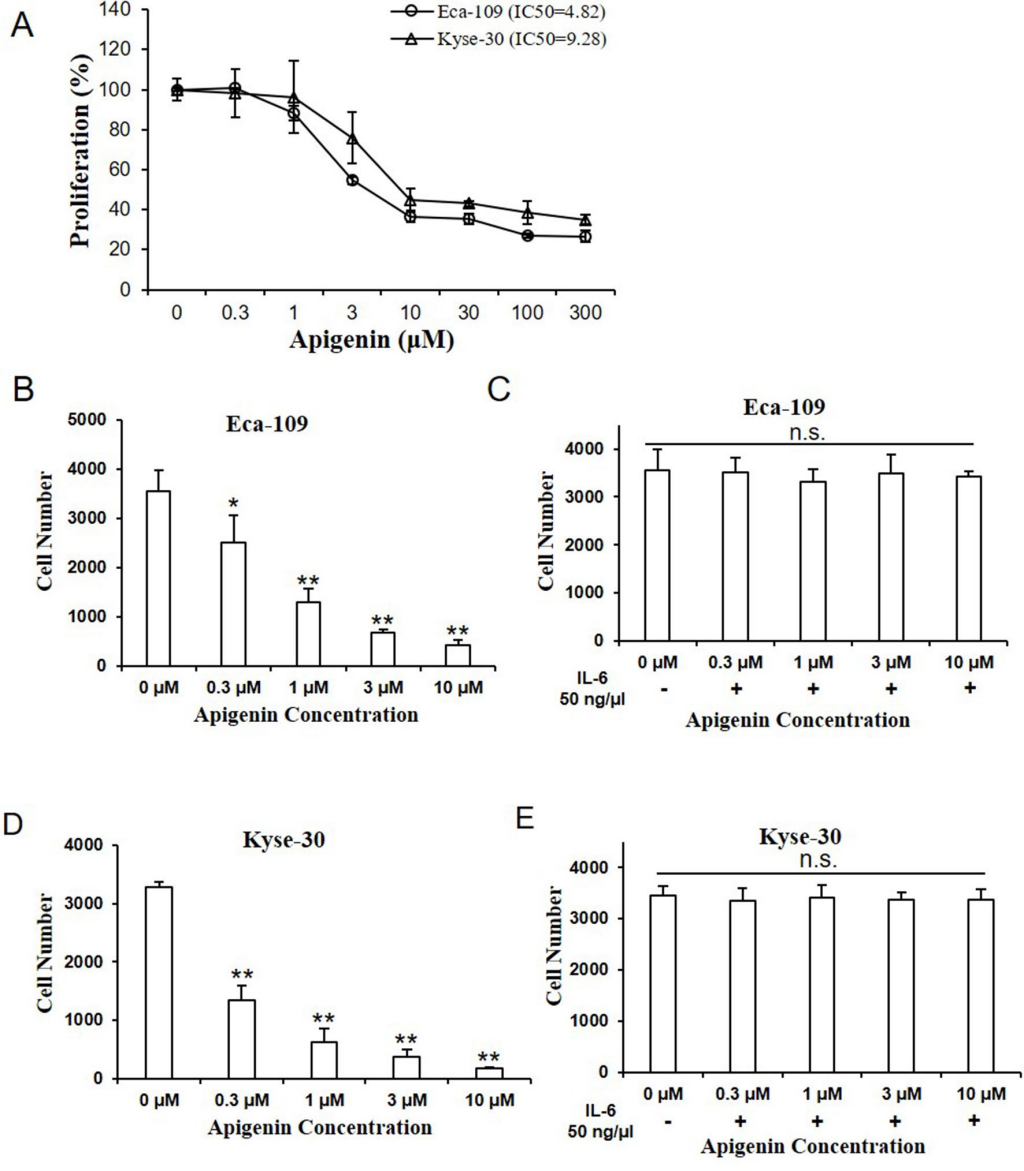
Apigenin inhibited cell proliferation in human esophagus cancer cells. **(A)** The cell proliferation rate of Eca-109 and Kyse-30 cells treated with indicated concentrations of apigenin, as determined by CCK-8 assay. **(B)** The cell number of Eca-109 and **(D)** Kyse-30 cells treated with apigenin (0, 0.3, 1, 3, and 10 µm). **(C)** The cell number of Eca-109 and **(E)** Kyse-30 cells pretreated with 50 ng/µl IL-6 for 6 h, prior to the addition of the indicated concentrations of apigenin. *P < 0.05 and **P < 0.01 indicate significant difference as compared to the corresponding control (*n* = 5).

We examined the effect of apigenin on cell proliferation by CCK-8 assay, with the relative cell proliferation rate (%) calculated using data from control group as 100%. As shown in **Figure 2A**, apigenin significantly inhibited cell proliferation rate dose-dependently in both Eca-109 and Kyse-30 cells. The IC_50_ values of apigenin in Eca-109 and Kyse-30 cell were 4.82 and 9.28 µm, respectively. To determine the cytotoxicity of apigenin, cells were treated with various concentrations of apigenin, and cell numbers were counted. As shown in **Figure 2B, D**, treatment of 0.3, 1, 3, and 10 µm of apigenin caused a significant 30%, 73%, 80%, and 89% reductions of cell growth in Eca-109 cells, respectively (**Figure 2B**), as well as 62.5%, 81.2%, 90%, and 95.8% reductions of cell growth in Kyse-30 cells, respectively (**Figure 2D**). Pretreating cells with 50 ng/µl of IL-6 for 6 h prior to the addition of apigenin completely reversed apigenin-induced changes (**Figure 2C, E**).

### Apigenin Induced Apoptosis in Esophagus Cancer Cells

**Figure 3:**
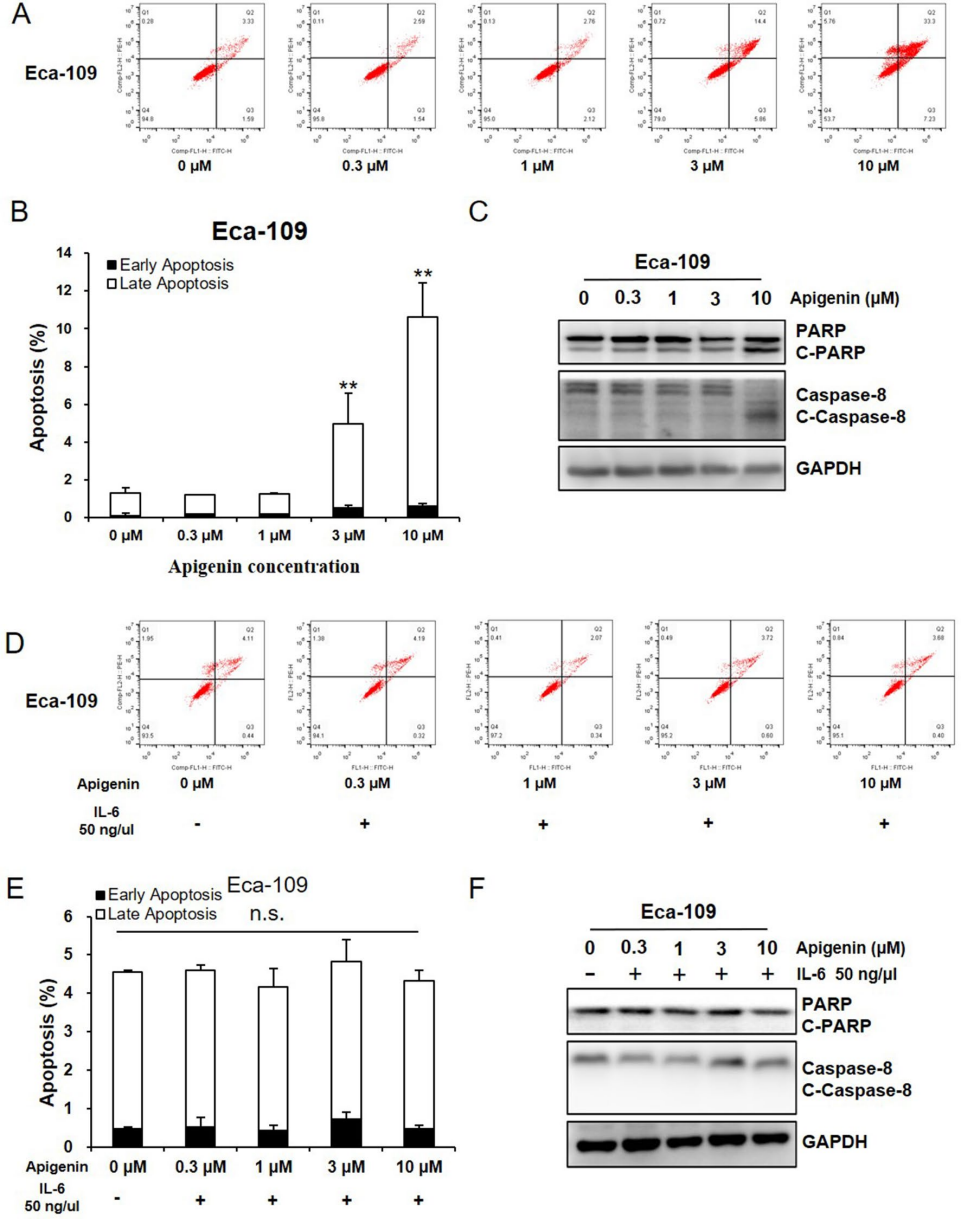
Apigenin induced apoptosis in Eca-109 cell. **(A**-**C)** Cells were treated with the indicated concentrations of apigenin, and the apoptosis was detected by FCM annexin V/PI staining. The protein expression levels were examined by Western blot, and GAPDH was used as loading control. **(D**–**F)** Cells were pretreated with 50 ng/µl IL-6, prior to the addition of indicated concentrations of apigenin, and the apoptosis was detected by FCM annexin V/PI staining. The protein expression was examined by Western blot. **(A**, **D)** the representative charts, **(B**, **E)** the quantified data, and **(C**, **F)** the Western blot results. *P < 0.05 and **P < 0.01 indicate significant difference as compared to the corresponding control (*n* = 3).

We next evaluated the effect of apigenin in cell apoptosis by flow cytometry (FCM). As shown in **Figure 3A, B**, treating Eca-109 cells with 1, 3, and 10 µM of apigenin increased early apoptosis (annexin V+/PI−) from 0.3% (control) to 0.5%, 1%, and 1.5%, respectively, and increased late apoptosis (annexin V+/PI+) from 1.6% (control) to 1.7%, 4%, and 9.5%, respectively. To understand the underlying mechanisms, we measured the apoptosis-related proteins by Western blotting analysis. Treating Eca-109 cells with 1, 3, and 10 µM of apigenin increased the apoptosis marker cleaved PARP (C-PARP) from 3% (control) to 15%, 36%, and 49%, respectively, and increased the apoptosis execution-phase marker, cleaved caspase-8 (C-caspase-8), from 12% (control) to 20%, 45%, and 83%, respectively (**Figure 3C**). Similar results were also found in the Kyse-30 cells (**Figure S1A–C**). In addition, pretreating Eca-109 cells with 50 ng/µl of IL-6 for 6 h prior to the addition of indicated concentrations of apigenin completely blocked apigenin-induced cell apoptosis (**Figure 3D, E**), as well as the expression of apoptosis markers (**Figure 3F**). Similar results were also found in the Kyse-30 cells (**Figure S1D–F**). These results suggested the possibility that apigenin-induced cell apoptosis works through IL-6 in esophagus cancer cells.

### Apigenin Inhibited Angiogenesis and VEGF Expression in Esophagus Cancer Cells

**Figure 4:**
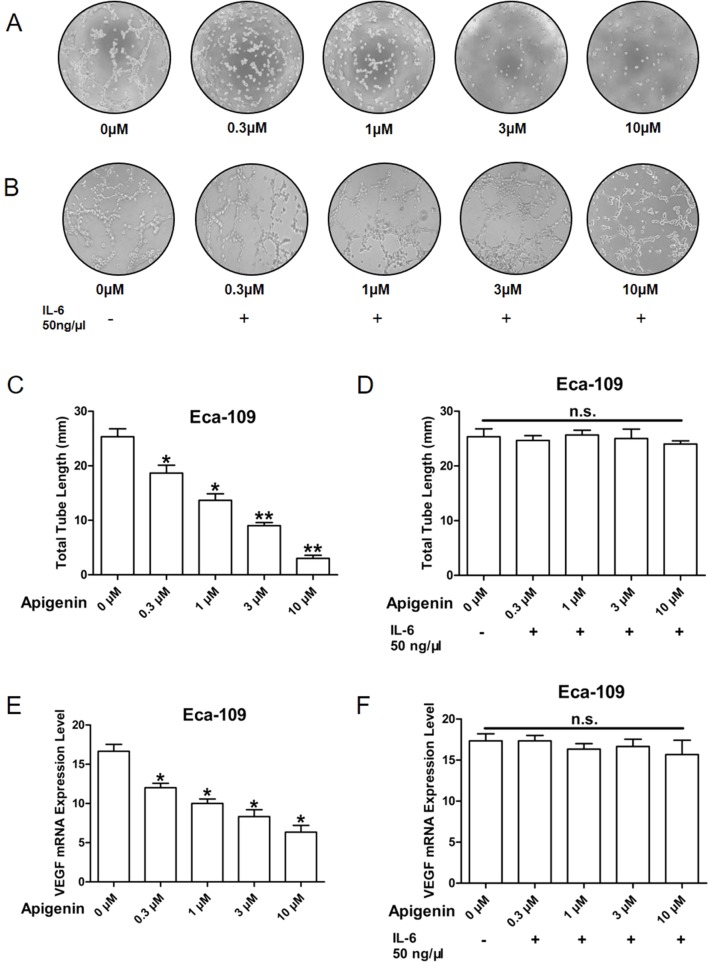
Apigenin inhibited angiogenesis in Eca109 cell. The CM was collected from Eca-109 cells cultured in serum free medium overnight, without **(A**, **C**, **E)** or with **(B**, **D**, **F)** the pretreatment of 50 ng/µl IL-6, prior to the addition of different concentrations of apigenin. The serum-reduced media were collected and stored at –20°C for later use. Tube formation assay was conducted as described in Methods section. The HUVECs were trypsinized, counted, and resuspended in EBM-2 basic medium, and then they were mixed with an equal volume of the CM, and tube formation was determined. The total lengths of the tubes in each well were measured using CellSens Standard software. **(A**, **B)** The representative picture of tube formation; **(C**, **D)** quantified data; **(E**, **F)** VEGF expression level as determined by quantitative PCR. *P < 0.05 and **P < 0.01 indicate significant difference as compared to the corresponding control (*n* = 3).

Angiogenesis is the essential process for tumor development (Chappell et al., 2019). Here we used tube formation assay to measure the activity of angiogenesis. It is well documented that HUVECs maintained in EBM-2 basic medium are not capable of tube formation; however, when incubated in conditioned medium (CM) prepared from tumor cells, tube formation activity is observed. We therefore evaluated whether CM from apigenin-treated Eca-109 and Kyse-30 cells could inhibit tube formation. As shown in **Figure 4A**, CM collected from Eca-109 cells treated with 0.3, 1, 3, and 10 µm of apigenin produced significant and dose-dependent reduction in tube formation activity. Furthermore, pretreating Eca-109 cells with 50 ng/µl of IL-6 prior to the addition of apigenin completely blocked the reduction in tube formation activity (**Figure 4B**). Apigenin treatment produced significant reduction in the total tube length (**Figure 4C**), and pretreatment with 50 ng/µl IL-6 completely blocked apigenin-induced reduction (**Figure 4D**). Similar results were also observed in Kyse-30 cells (**Figures S2A–D**).

VEGF plays a key role in tumor-induced angiogenesis. Therefore, we evaluated the effect of apigenin on the gene expression of VEGF in Eca-109 and Kyse-30 cells. As shown in **Figure 4E**, 0.3, 1, 3, and 10 µM of apigenin produced significant 28%, 40%, 50%, and 62% reductions in VEGF expression, respectively, in Eca-109 cells. Pretreatment of Eca-109 cells with 50 ng/µl of IL-6 completely blocked the reductions (**Figure 4F**). Similar results were also found in the Kyse-30 cells (**Figures S2E, F**). Taken together, these results raised the possibility that apigenin may inhibit tumor angiogenesis and VEGF expression through IL-6.

### Apigenin Treatment Inhibited the Growth of Esophagus Tumor *in vivo* in Nude Mice Subcutaneously Xenografted With Eca-109 Cells

**Figure 5:**
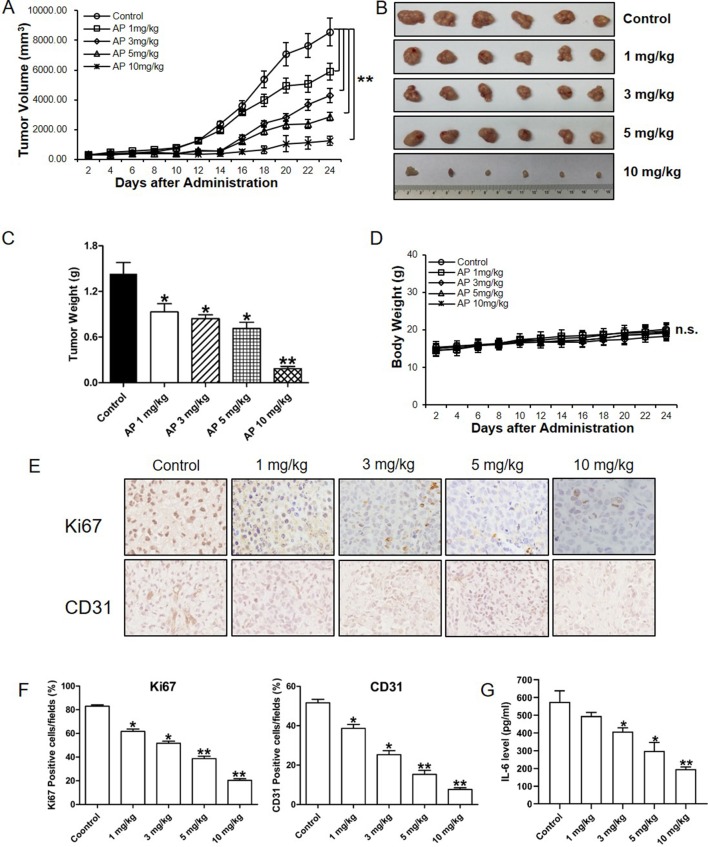
Apigenin inhibited the growth of esophagus cancer in nude mice xenografted with Eca-109 cells. Nude mice were injected subcutaneously with Eca-109 cells, randomly divided into five groups, and treated by intraperitoneal injection with the following regimens: vehicle alone (0.9% saline) and apigenin (1, 3, 5, and 10 mg/kg) every other day. **(A)** The tumor volume, **(B)** photos of the tumors, **(C)**, tumor weight, and **(D)** body weight. The collected tumor tissues were analyzed by quantitative PCR and immunohistochemical analysis. **(E)** The immunohistochemical Ki67 and CD31 staining, **(F)** quantified positive cells, and **(G)** IL-6 expression level as detected by ELISA. The values presented are the means ± SD. *P < 0.05 and ** *P* < 0.01 indicate significant difference from the corresponding control (*n* = 6).

To study the *in vivo* effect of apigenin treatment in esophagus tumor, a preclinical animal model of nude mice subcutaneously xenografted with Eca-109 cells was generated. As shown in **Figure 5A**, apigenin treatment produced dose-dependent inhibition in tumor growth. Treatment with 1, 3, 5, and 10 mg/kg of apigenin produced significant 35.12%, 41.28%, 50.23%, and 87.21% reductions, respectively, in tumor weight on day 24 after initiation of treatment (**Figure 5B, C**). Furthermore, there was no significant loss of mice body weight in the apigenin treatment groups, suggesting that the indicated dose of apigenin might not cause severe toxicity in nude mice (**Figure 5D**). To study the association of IL-6 in apigenin-mediated inhibition *in vivo*, the tumor tissues were analyzed by immunohistochemical staining. Apigenin significantly inhibited the expression levels of Ki67 (the cell proliferation necessary protein) and CD31 (the angiogenesis key protein) (**Figure 5E, F**). Importantly, apigenin significantly inhibited the level of IL-6 in the xenograft tumor tissues (**Figure 5G**).

## Discussion

In the present study, we reported that apigenin inhibited IL-6 transcription and gene expression in esophagus cancer cells. We also provided *in vitro* and *in vivo* evidence suggesting that its antitumor activities work at least in part through IL-6. Therefore, we hypothesize that inhibiting IL-6 transcription is a new mechanism by which apigenin exhibits its antitumor activity in esophagus cancer.

At present, two monoclonal antibodies against IL-6 are tested in various preclinical studies for cancer treatments. Tocilizumab was tested in ovarian cancer (Dijkgraaf et al., 2015) and siltuximab in multiple myeloma (Voorhees et al., 2013; Orlowski et al., 2015), ovarian cancer (Coward et al., 2011), and prostate cancer (Dorff et al., 2010). To our knowledge, apigenin is the first reported small molecule IL-6 transcription inhibitor. Interleukin 6 is overexpressed in a significant portion of cancers. The potential therapeutic effect of apigenin for the treatment of esophageal and other IL-6–overexpressing cancers requires further investigations.

At present, it is not known how apigenin could inhibit the transcription activity of IL-6. Interleukin 6 gene has three transcription start sites and three TATA boxes. The three transcription start sites contain two glucocorticoid response elements (GREs, -557 to -552 and -466 to -461) and one activator protein 1 (AP-1)–binding site (-283 to -277) (Edbrooke et al., 1989; Isshiki et al., 1990). The GREs and AP-1 sequences have high similarity and only one difference in two nucleotides (Grassl et al., 1999). The possibility that apigenin regulated the transcription activity of IL-6 *via* interaction with these transcription start sites and GREs, AP-1, and TATA boxes remains to be determined.

In this study, our results indicated that apigenin could regulate multiple carcinogenesis pathways in esophagus cancer cells. These include cell proliferation, angiogenesis, and apoptosis *via* regulating the expression level of PARP, caspase-8, VEGF, and their downstream factors. As IL-6 plays multiple functions in carcinogenesis, these results are consistent with what to be expected as an IL-6 inhibitor. Interleukin 6 has also been shown to play key functions in multidrug resistance *via* JAK, STAT3, PI3K/Akt, and Ras-MAPK signal pathways (Ghandadi and Sahebkar, 2016; Zang et al., 2017), tumor cell migration (Che et al., 2019), invasion (Cao et al., 2017), and other pathways of carcinogenesis. The potential functions of apigenin against multidrug resistance, migration, and invasion in esophagus cancer require further investigations.

Our results are also consistent with previous reports that apigenin inhibited tumor growth *via* multiple signal pathways. For instance, apigenin inhibited tumor metastasis through AKT/p70S6K1/MMP-9 signaling in ovarian cancer (Fang et al., 2005), suppressed tumor angiogenesis through HIF-1/VEGF signaling (Fang et al., 2007), inhibited tumorigenesis *via* WNT/β-catenin signaling (Ozbey et al., 2018), suppressed lung cancer progression *via* Akt/Snail/Slug signaling (Chang et al., 2018), inhibited the development and progression of prostate cancer *via* insulinlike growth factor signaling (Babcook and Gupta, 2012), and inhibited cell growth, metastasis, and tumor angiogenesis in human lung cancer cells through PI3K/AKT/mTOR/p70S6K1 and AKT/p70S6K1/MMP-9 signaling pathways (Meng et al., 2006; He et al., 2012). The potential cross-talks between the IL-6 signal pathways and the PI3K/AKT/mTOR/p70S6K1 and AKT/p70S6K1/MMP-9 signaling pathways remain to be evaluated.

Natural and innoxious agents have long proposed to inhibit or prevent human cancer development. There are several reviews of the epidemiological and dietary studies of apigenin in cancers. Results from an epidemiological study evaluating the polyphenol consumption and human cancer risk have supported the protective effects of certain food items and polyphenols in reducing cancer risk (Yang et al., 2001). There is also a report suggesting that sustained long-term treatment with a flavonoid mixture could reduce the recurrence rate of colon neoplasia in patients with resected cancer (Hoensch et al., 2008). However, the factors and molecules that contribute to the protective effects are still not well understood. Our daily diets are rich in flavonoids, and apigenin is one of the most common flavonoids, with proved anticancer activity (Bhattacharya et al., 2018; Chang et al., 2018; Li et al., 2018; Xia et al., 2018; Xu et al., 2018). Therefore, the potential anticancer activity of apigenin and its applications warrant further investigations.

In addition to tumorigenesis, IL-6 also plays important roles in inflammation, hematopoiesis, and other physiological and pathological responses. Since IL-6 has diverse and multiple functions in different tissues, an inhibitor simply targeting at IL-6 would cause severe side effects. Nevertheless, as a small molecule IL-6 gene transcription inhibitor, the effect of apigenin on IL-6 is likely to be more tissue and tumor type specific and therefore may have less toxic side effects.

At the lethal dose of 50% (LD_50_) in rat equal to 727.76 mg/kg, apigenin appears to be a nontoxic and safe molecule. In our study, apigenin (1 mg/kg, intraperitoneal injection for 24 days) significantly inhibited the growth of Eca-109 xenograft tumors *in vivo* in nude mice without loss of body weight, suggesting that it may be a relatively safe drug. In addition, no significant changes in other health characteristics including behaviors, water intake, and food intake were observed by the apigenin treatment. The drug toxicity evaluation and potential applications of apigenin for esophageal and other cancer treatments require further investigations.

## Data Availability

All datasets generated for this study are included in the manuscript/**Supplementary files**.

## Ethics Statement

The animal study was reviewed and approved by Institutional Review Committees of Zhengzhou University.

## Author Contributions

J-GQ, LW, W-JL, L-HW, Z-KX, and WW performed experiments. B-HJ, Y-XH, L-ZL, and MC-ML designed the studies. J-FW, E-JZ, F-MZ, and X-BJ wrote the manuscript. All authors read and approved the final manuscript.

## Funding

This work was supported by the China Postdoctoral Science Foundation Grant (no. 2017M622378), the National Natural Science Foundation of China (no. 81803197, no. 81903174); the National Institutes of Health grants (no. R01ES020868, no. R01CA193511, no. R01CA232587), the Science and Technology Research Project of Henan province (no. 192102310101), American Cancer Society Research Scholar (no. NEC-129306), and Postdoctoral Research Grant in Henan Province (no. 001803002).

## Conflict of Interest Statement

The authors declare that the research was conducted in the absence of any commercial or financial relationships that could be construed as a potential conflict of interest.

## Supplementary Material

Figure S1 Apigenin induced apoptosis in Kyse-30 cell. **(A**–**C)** Cells were treated with the indicated concentrations of apigenin, and the apoptosis was detected by FCM annexin V/PI staining. The protein expression levels were examined by Western blot, and GAPDH was used as loading control. **(D**-**F)** Cells were pretreated with 50 ng/µl IL-6, prior to the addition of indicated concentrations of apigenin, and the apoptosis was detected by FCM annexin V/PI staining. The protein expression was examined by Western blot. **(A**, **D)** The representative charts, **(B**, **E)** quantified data, and **(C**, **F)** Western blot results. **P*< 0.05 and ***P*< 0.01 indicate significant difference as compared to the corresponding control (*n* = 3).

Figure S2 Apigenin inhibited angiogenesis and IL-6 prevented Apigenin mediated angiogenesis in Kyse-30 cell. Kyse-30 cells were cultured in serum free medium overnight, without **(A**, **C**, **E)** or with **(B**, **D**, **F)** the pretreatment of 50 ng/µl IL-6, prior to the addition of different concentrations of apigenin. The serum-reduced media were collected and stored at –20°C for later use. Tube formation assay was conducted as described in the Methods section. The HUVECs were trypsinized, counted, and resuspended in EBM-2 basic medium, and then they were mixed with an equal volume of the CM, and tube formation was determined. The total lengths of the tubes in each well were measured using CellSens Standard software. **(A**, **B)** The representative picture of tube formation, **(C**, **D)** quantified data, and **(E**, **F)** VEGF expression level as determined by quantitative PCR. **P*< 0.05 and ***P*< 0.01 indicate significant difference as compared to the corresponding control (*n* = 3).
