# Supplementary figures and images for: Apigenin Inhibits IL-6 Transcription and Suppresses Esophageal Carcinogenesis
Source: Front Pharmacol. 2019 Sep 11;10:1002. doi: 10.3389/fphar.2019.01002 (PMC6749068; doi:10.3389/fphar.2019.01002)

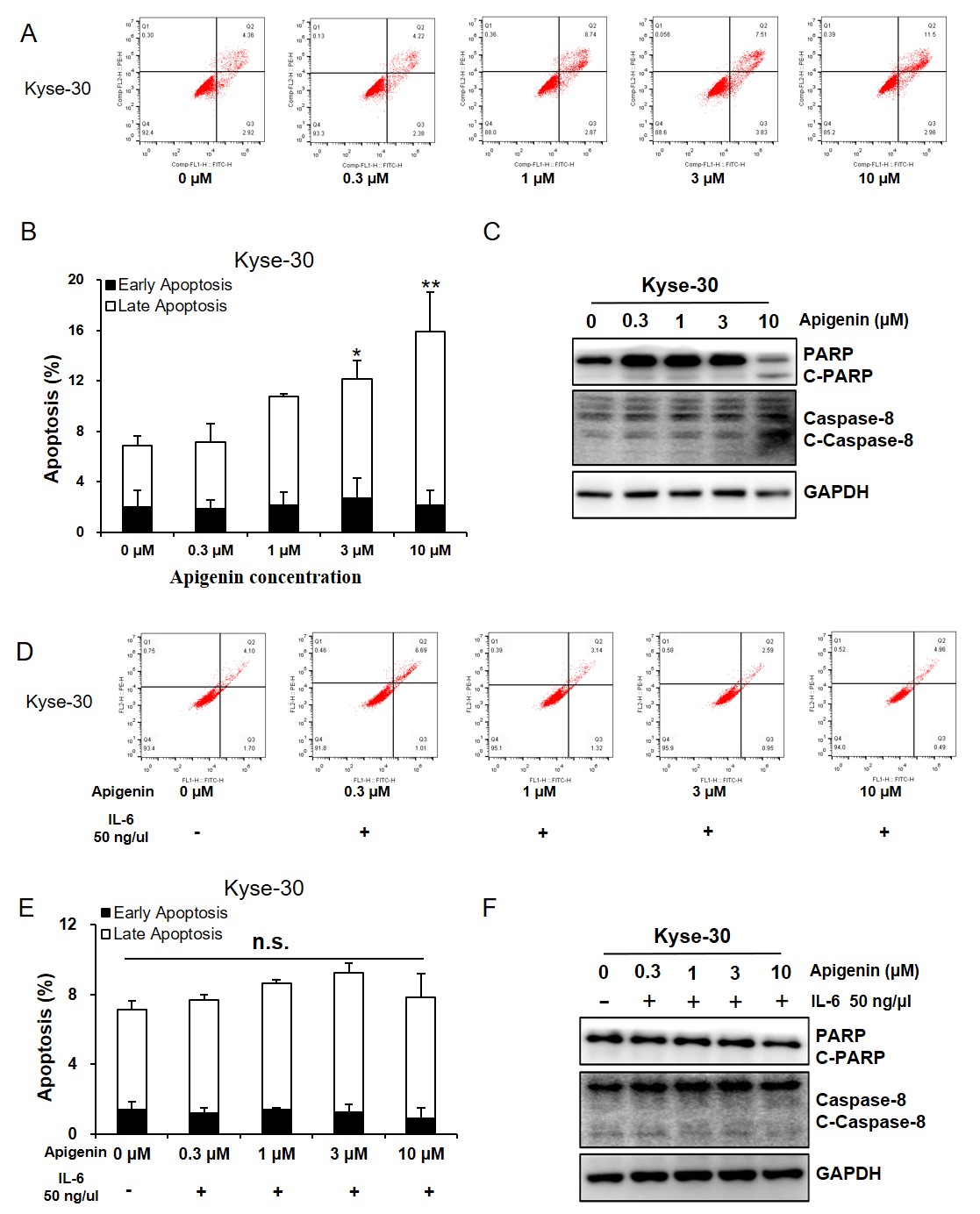

Supplement: Figure S1 — Apigenin induced apoptosis in Kyse-30 cell. (A–C) Cells were treated with the indicated concentrations of apigenin, and the apoptosis was detected by FCM annexin V/PI staining. The protein expression levels were examined by Western blot, and GAPDH was used as loading control. (D-F) Cells were pretreated with 50 ng/µl IL-6, prior to the addition of indicated concentrations of apigenin, and the apoptosis was detected by FCM annexin V/PI staining. The protein expression was examined by Western blot. (A, D) The representative charts, (B, E) quantified data, and (C, F) Western blot results. *P< 0.05 and **P< 0.01 indicate significant difference as compared to the corresponding control (n = 3). [file Image_1.jpeg]

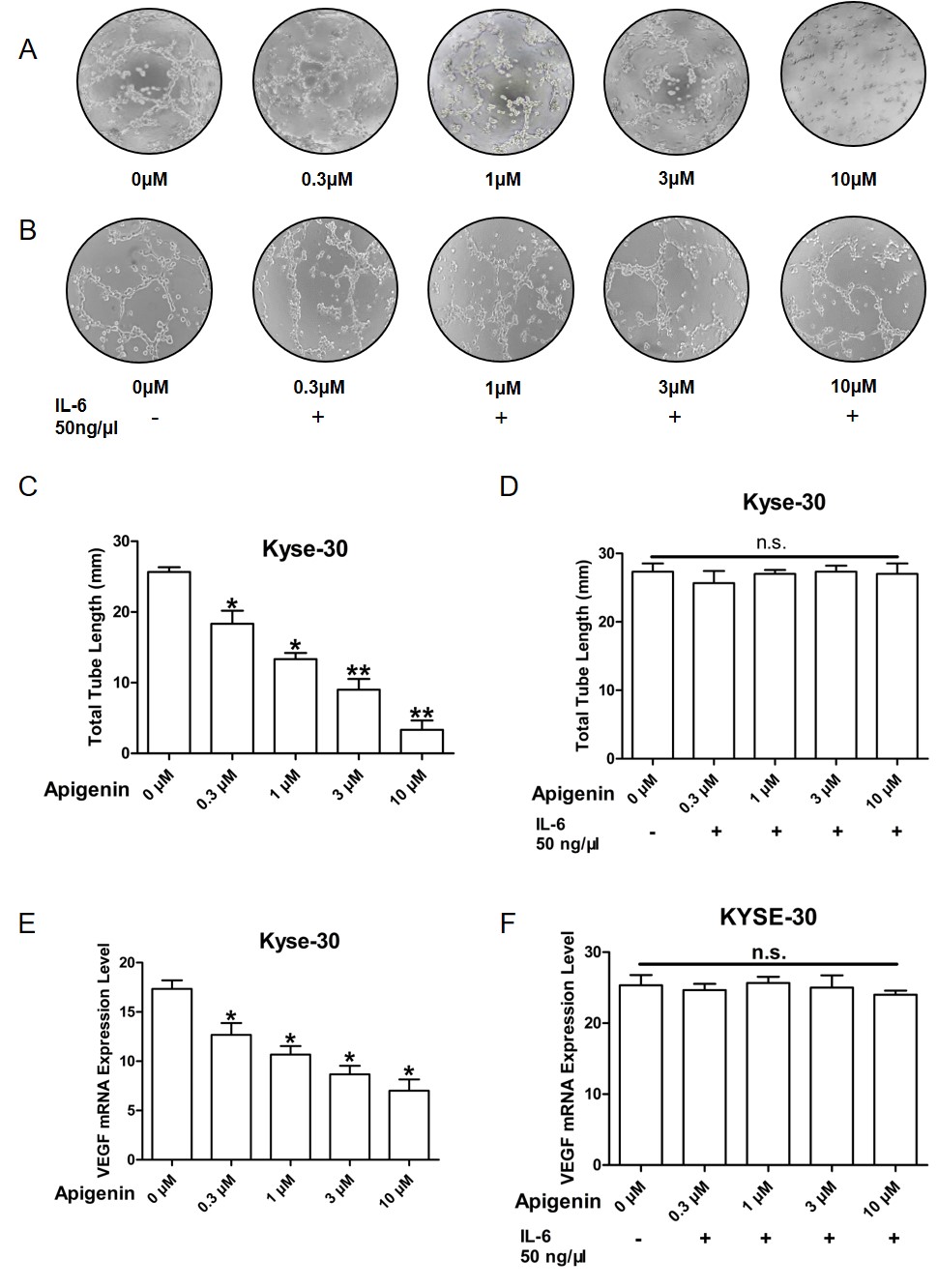

Supplement: Figure S2 — Apigenin inhibited angiogenesis and IL-6 prevented Apigenin mediated angiogenesis in Kyse-30 cell. Kyse-30 cells were cultured in serum free medium overnight, without (A, C, E) or with (B, D, F) the pretreatment of 50 ng/µl IL-6, prior to the addition of different concentrations of apigenin. The serum-reduced media were collected and stored at –20°C for later use. Tube formation assay was conducted as described in the Methods section. The HUVECs were trypsinized, counted, and resuspended in EBM-2 basic medium, and then they were mixed with an equal volume of the CM, and tube formation was determined. The total lengths of the tubes in each well were measured using CellSens Standard software. (A, B) The representative picture of tube formation, (C, D) quantified data, and (E, F) VEGF expression level as determined by quantitative PCR. *P< 0.05 and **P< 0.01 indicate significant difference as compared to the corresponding control (n = 3). [file Image_2.jpeg]

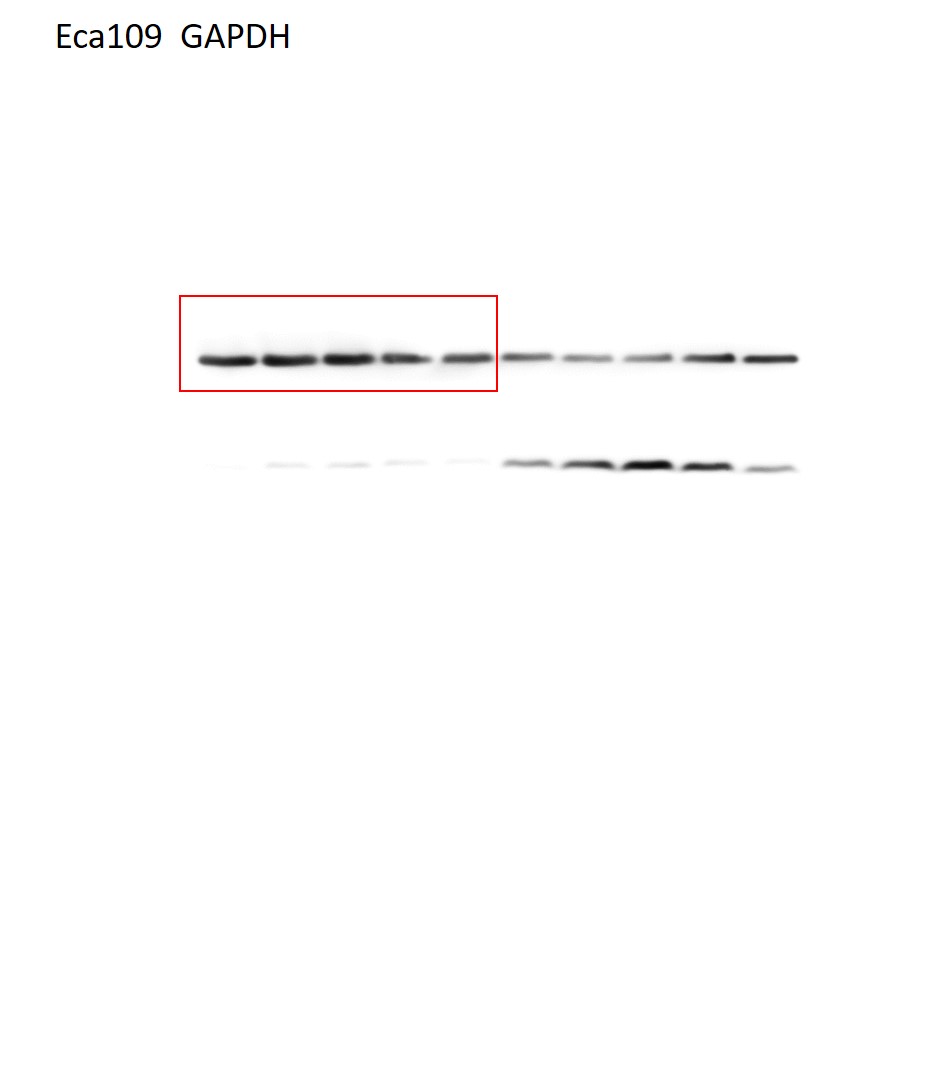

Supplement: Supplementary file 3 [file DataSheet_1.zip › Original picture of western blot/F1B-ECA109-GAPDH.jpg]

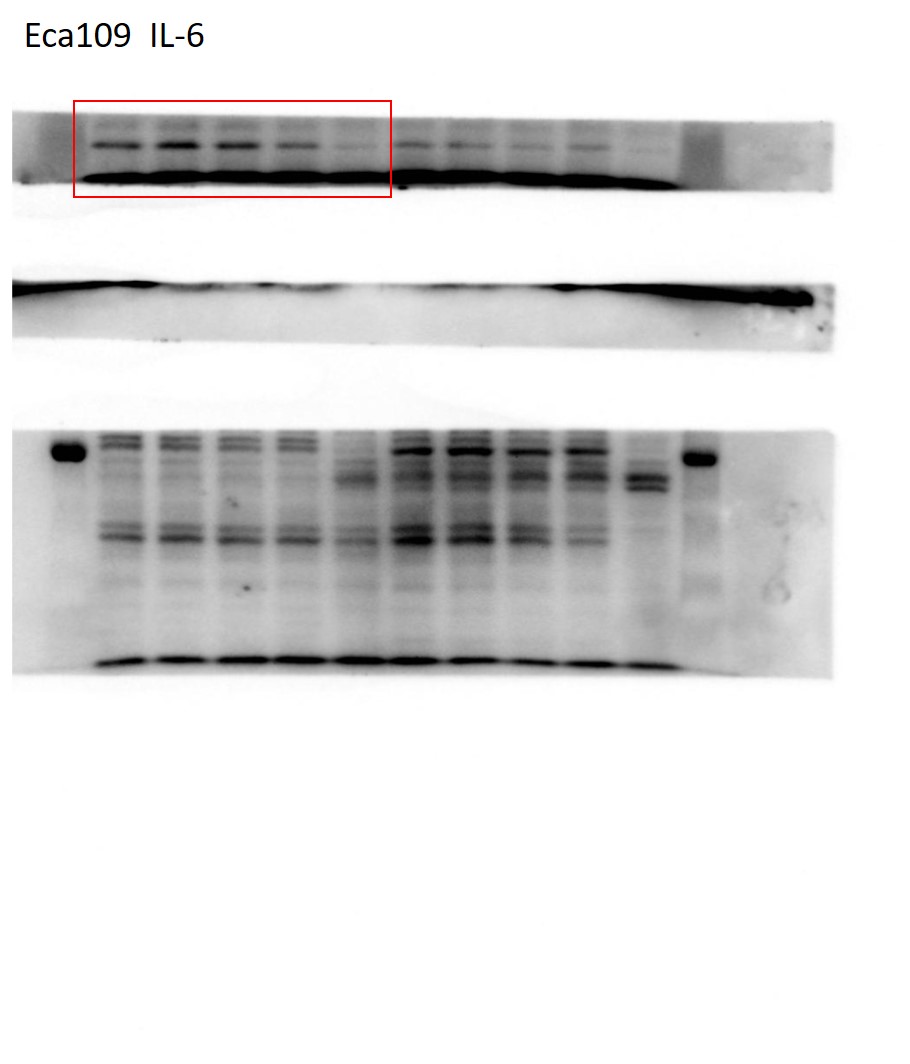

Supplement: Supplementary file 3 [file DataSheet_1.zip › Original picture of western blot/F1B-ECA109-IL6.jpg]

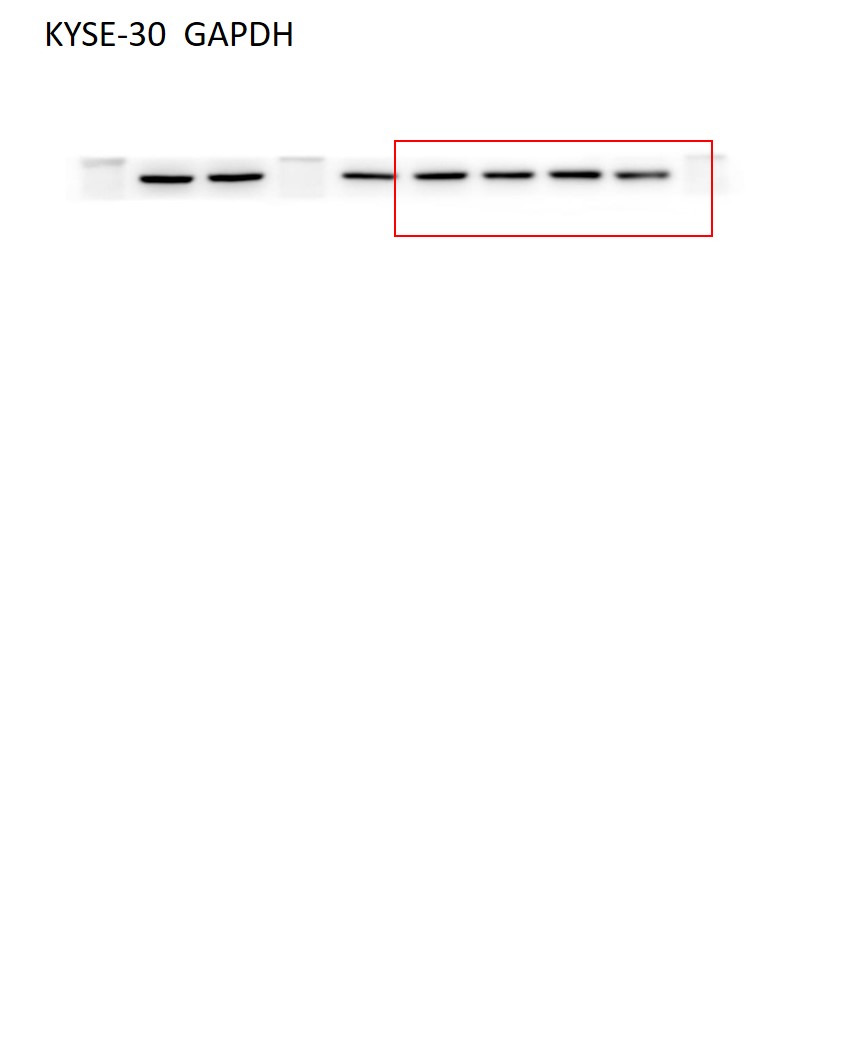

Supplement: Supplementary file 3 [file DataSheet_1.zip › Original picture of western blot/F1B-KYSE30-GAPDH.jpg]

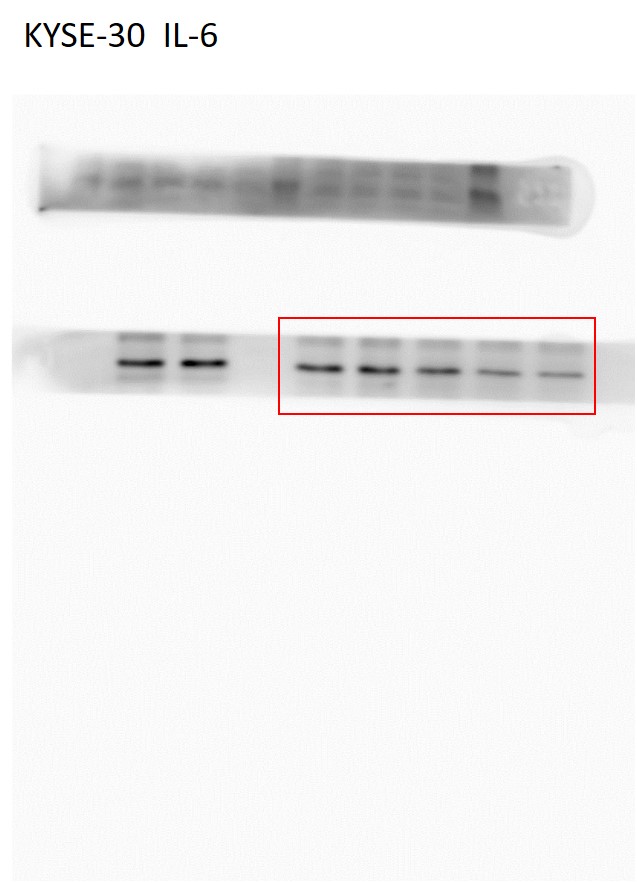

Supplement: Supplementary file 3 [file DataSheet_1.zip › Original picture of western blot/F1B-KYSE30-IL6.jpg]

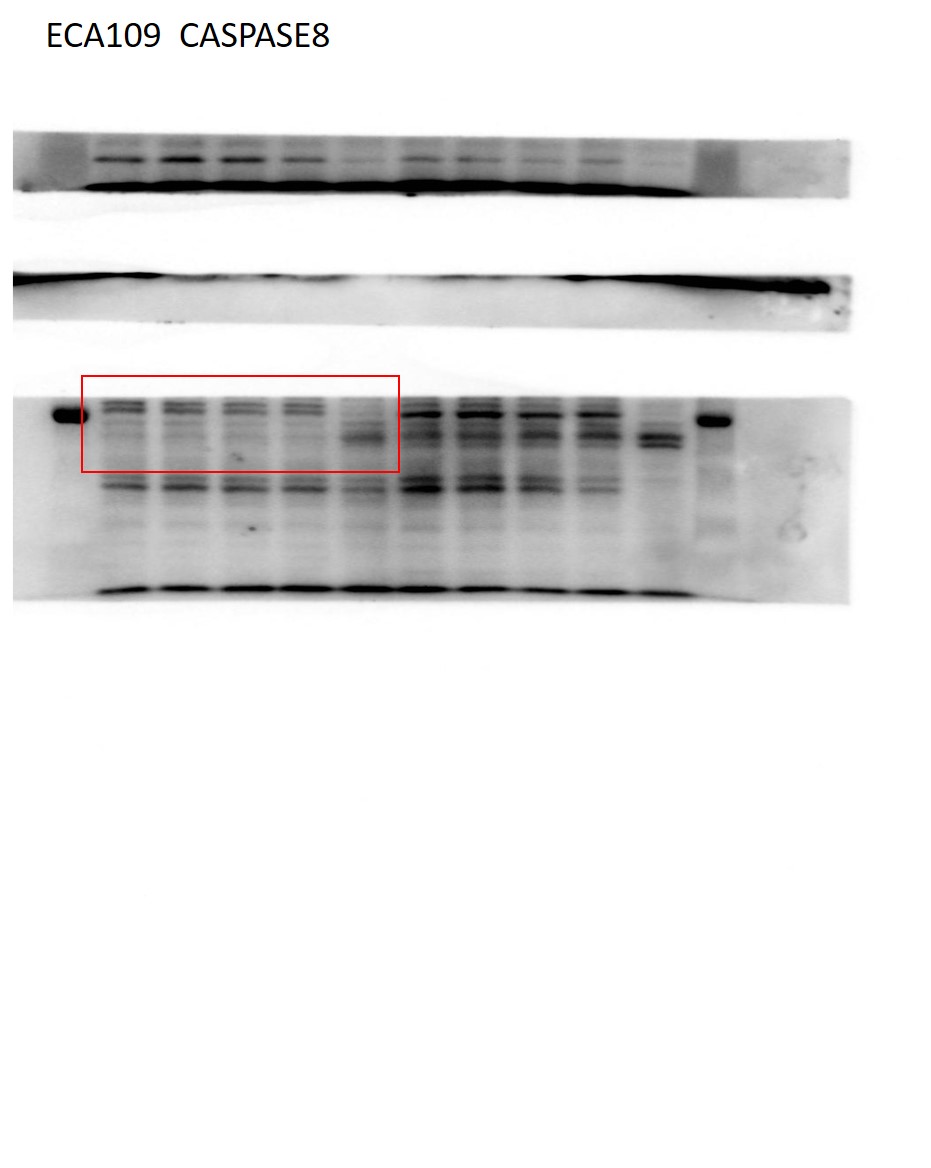

Supplement: Supplementary file 3 [file DataSheet_1.zip › Original picture of western blot/F3C-ECA109-CASPASE8.jpg]

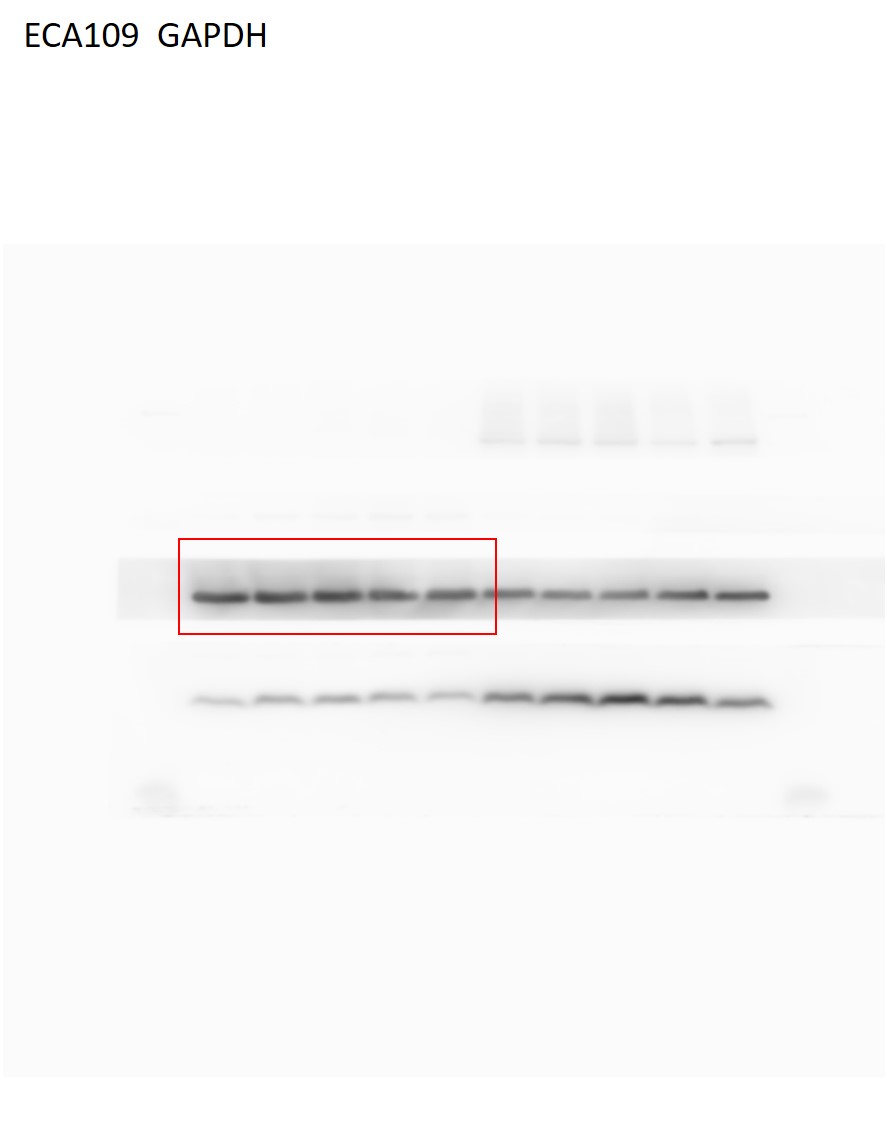

Supplement: Supplementary file 3 [file DataSheet_1.zip › Original picture of western blot/F3C-ECA109-GAPDH.jpg]

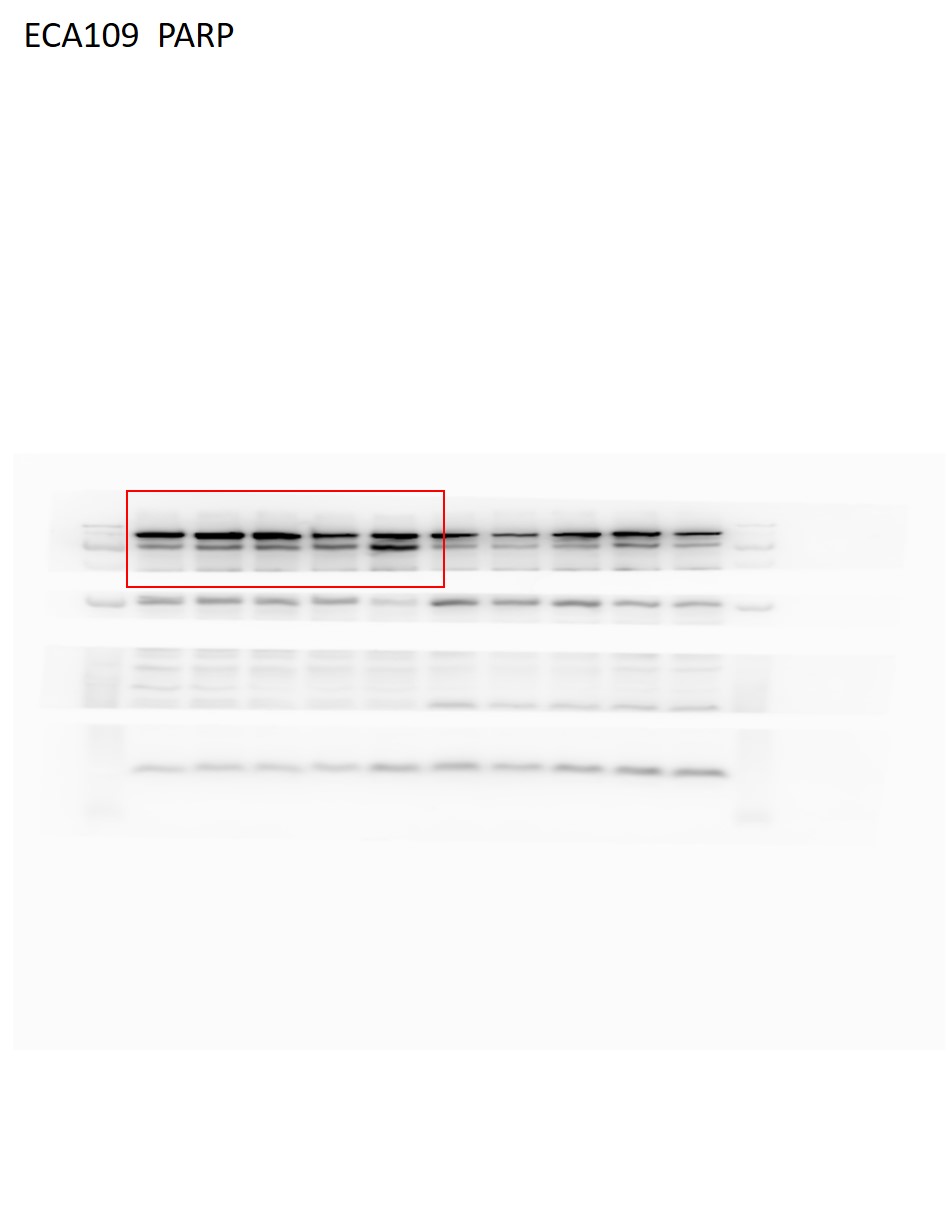

Supplement: Supplementary file 3 [file DataSheet_1.zip › Original picture of western blot/F3C-ECA109-PARP.jpg]

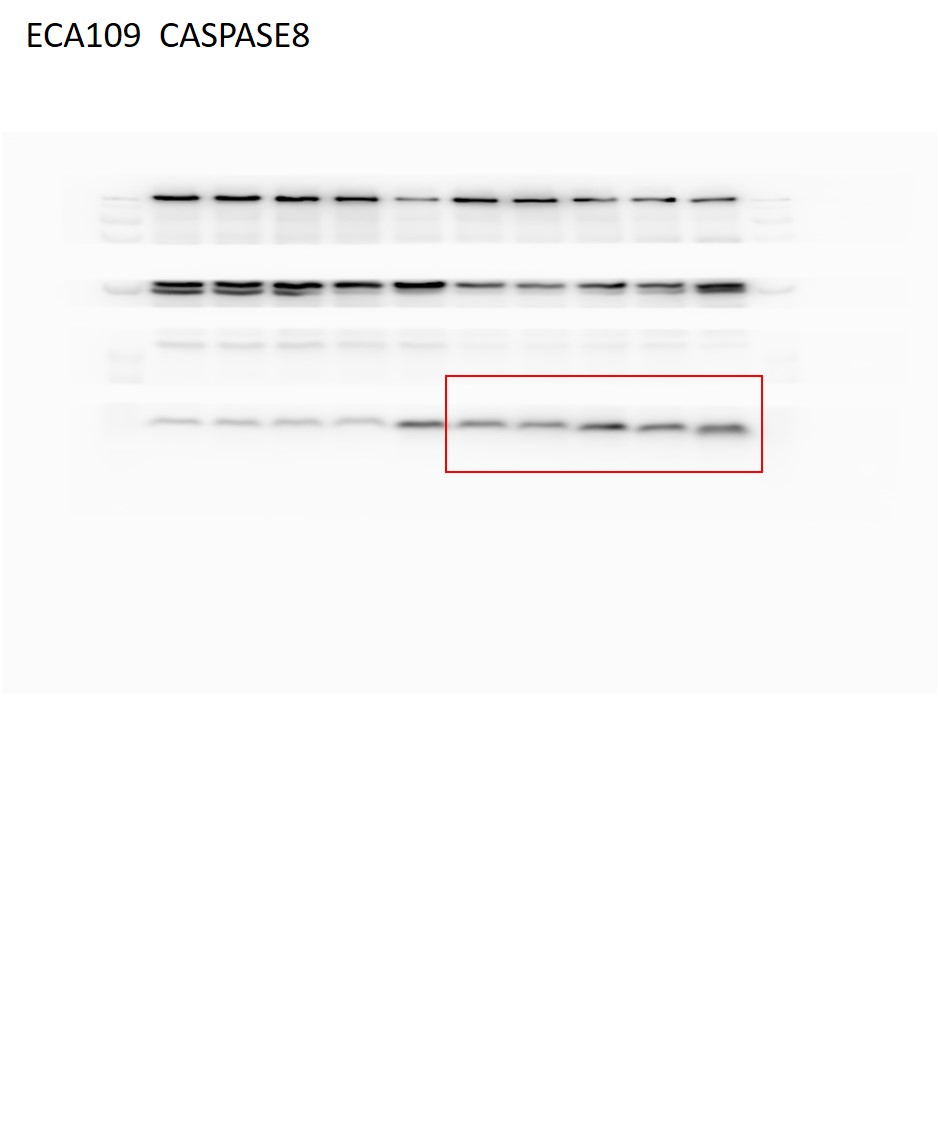

Supplement: Supplementary file 3 [file DataSheet_1.zip › Original picture of western blot/F3F-ECA109-CASPASE8.jpg]

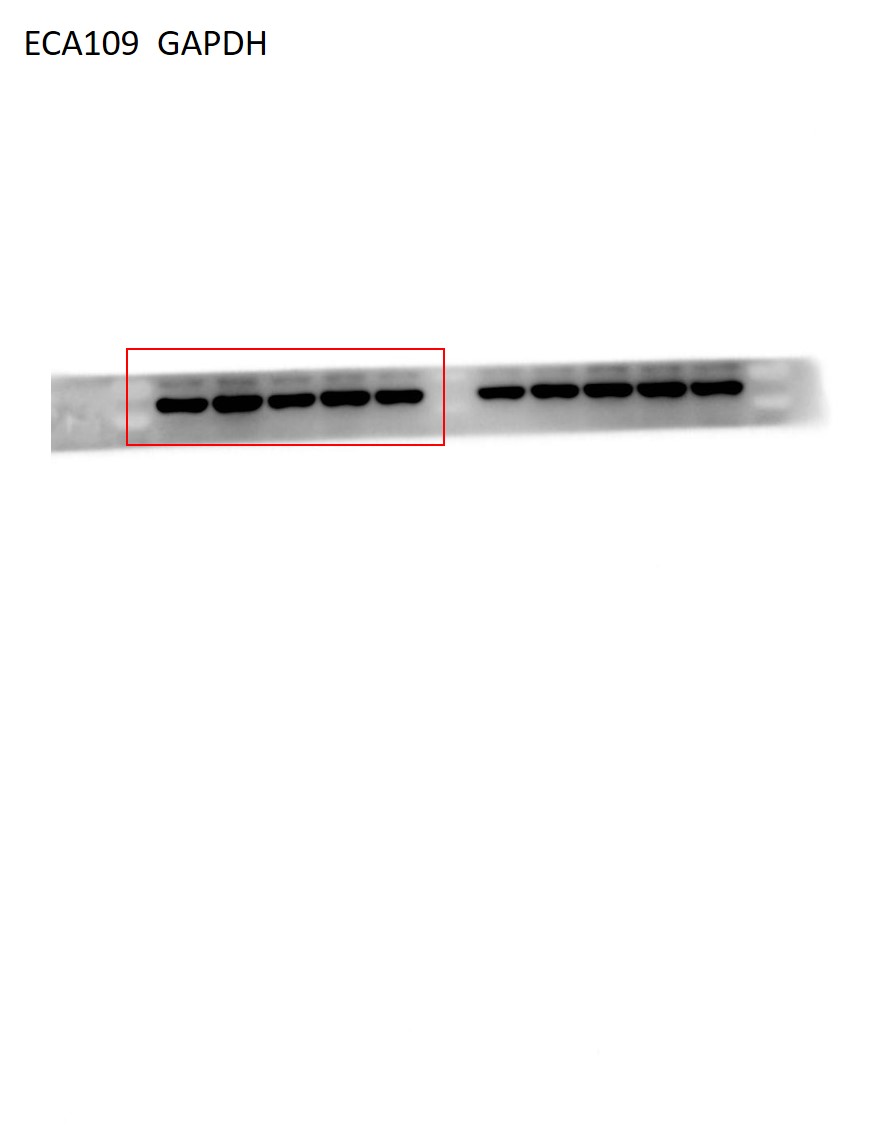

Supplement: Supplementary file 3 [file DataSheet_1.zip › Original picture of western blot/F3F-ECA109-GAPDH.jpg]

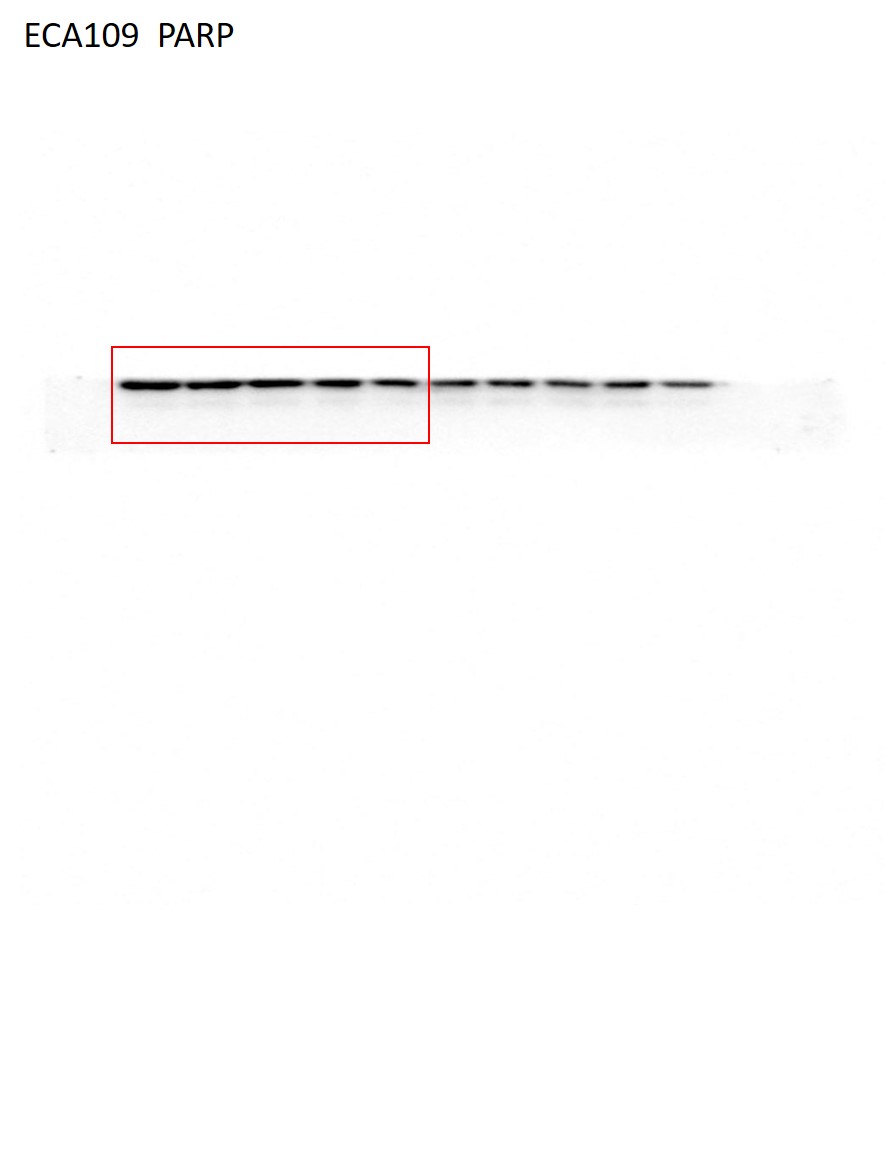

Supplement: Supplementary file 3 [file DataSheet_1.zip › Original picture of western blot/F3F-ECA109-PARP.jpg]

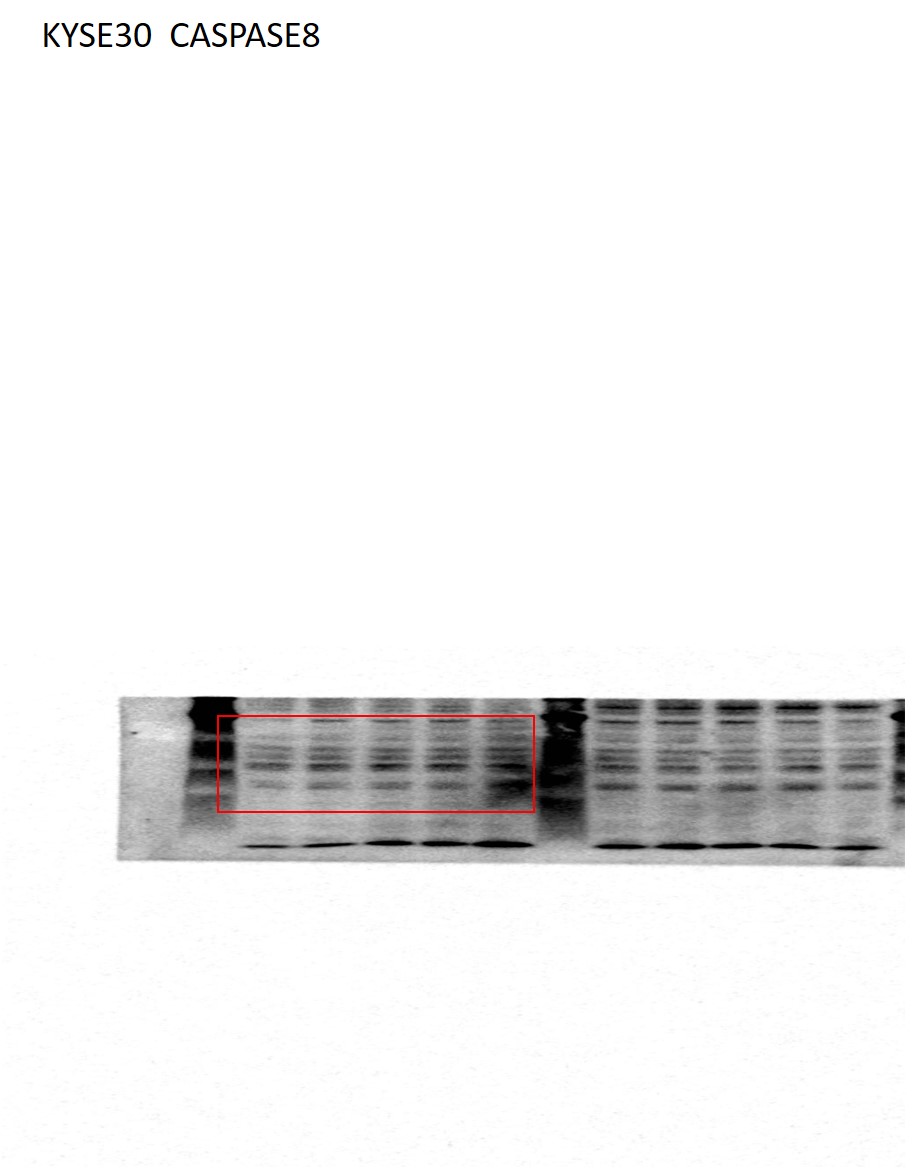

Supplement: Supplementary file 3 [file DataSheet_1.zip › Original picture of western blot/FS1C-KYSE30-CASPASE8.jpg]

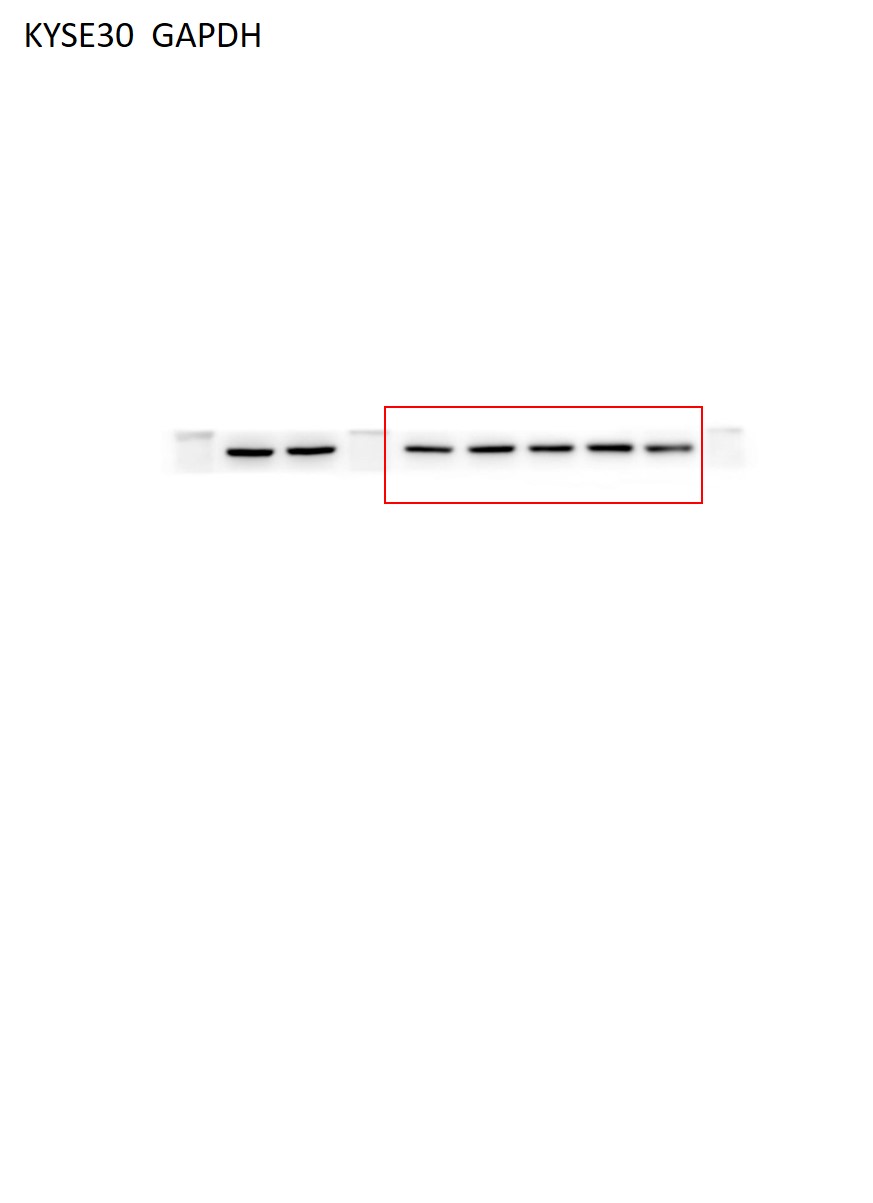

Supplement: Supplementary file 3 [file DataSheet_1.zip › Original picture of western blot/FS1C-KYSE30-GAPDH.jpg]

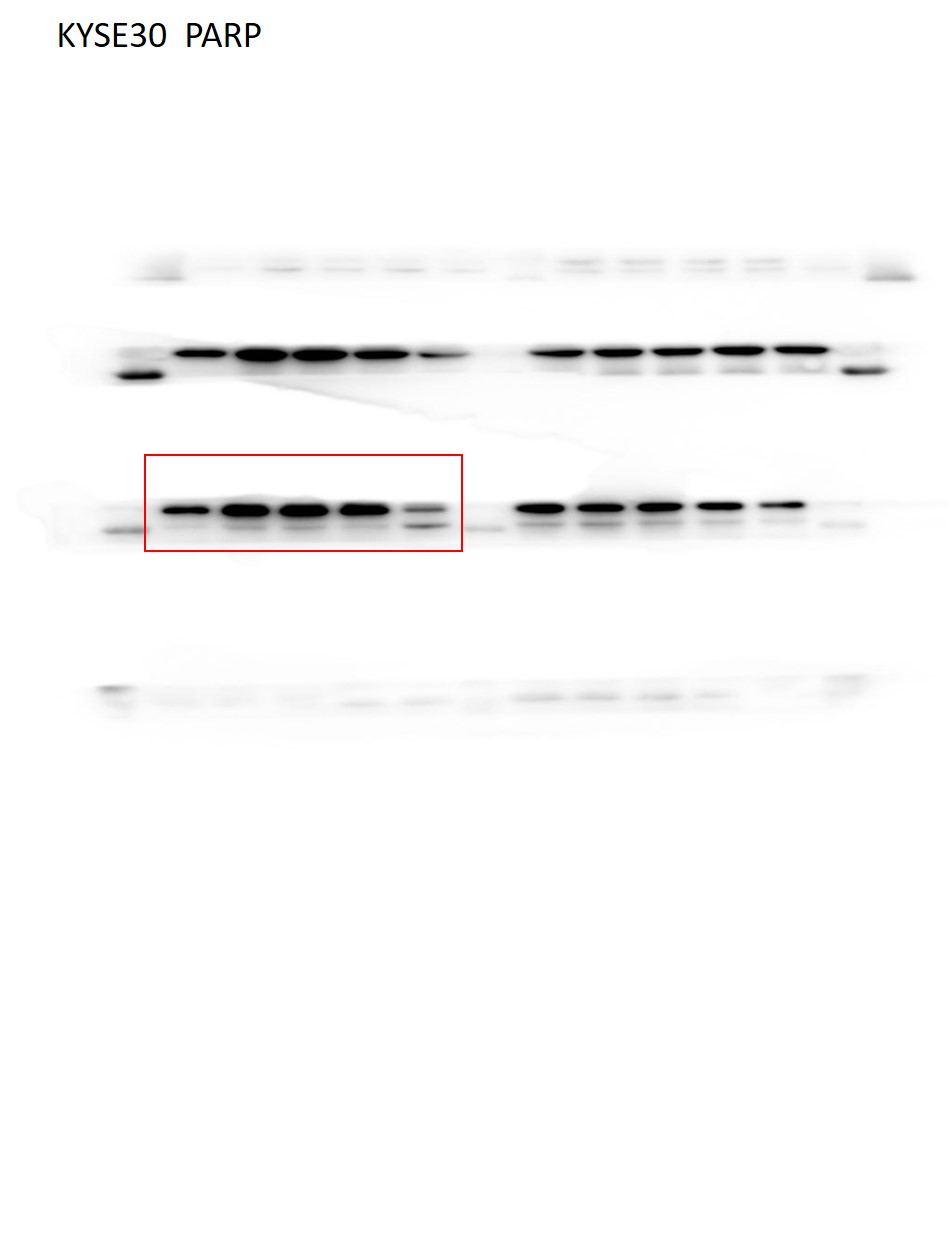

Supplement: Supplementary file 3 [file DataSheet_1.zip › Original picture of western blot/FS1C-KYSE30-PARP.jpg]

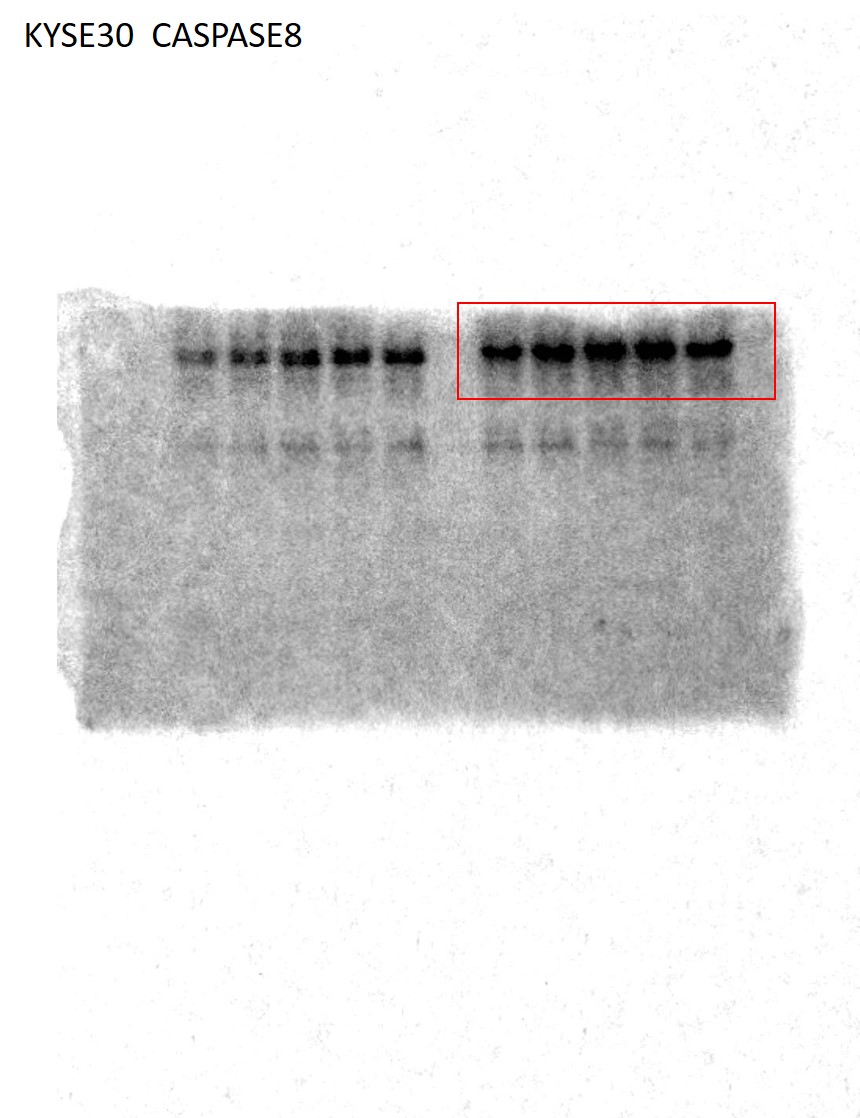

Supplement: Supplementary file 3 [file DataSheet_1.zip › Original picture of western blot/FS1F-KYSE30-CASPASE8.jpg]

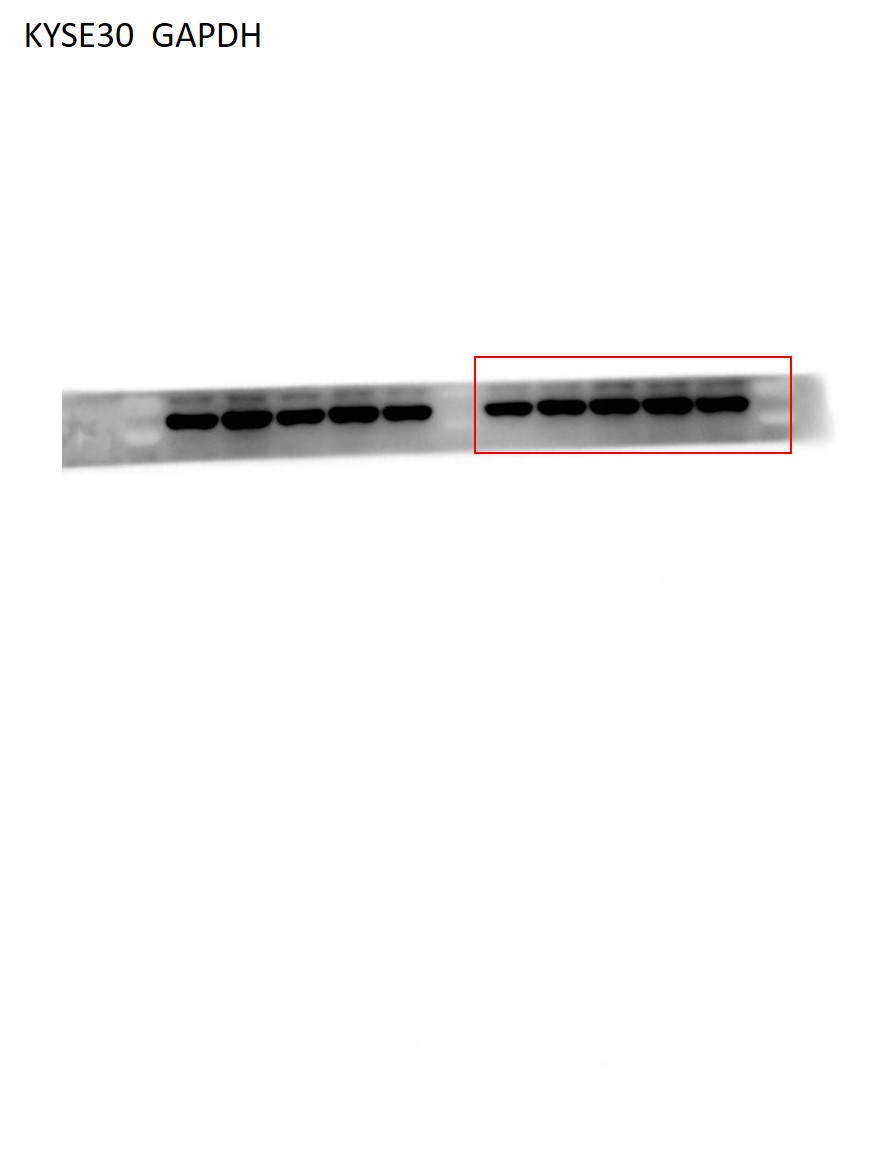

Supplement: Supplementary file 3 [file DataSheet_1.zip › Original picture of western blot/FS1F-KYSE30-GAPDH.jpg]

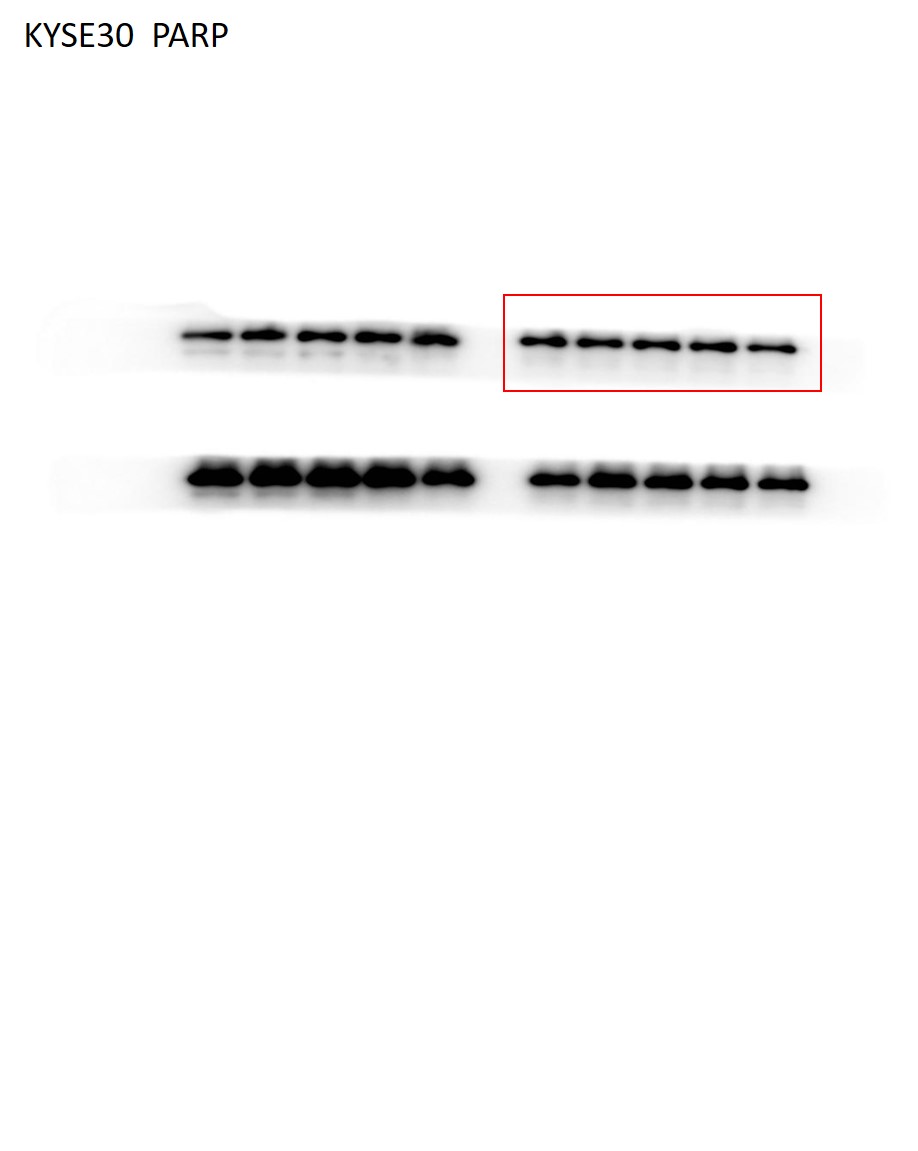

Supplement: Supplementary file 3 [file DataSheet_1.zip › Original picture of western blot/FS1F-KYSE30-PARP.jpg]
